# How Mixed Are Production‐Oriented Planted Forests? A Global Review and Classification Approach

**DOI:** 10.1111/gcb.71111

**Published:** 2026-09-18

**Authors:** Leticia Bulascoschi Cagnoni, Martin Weih, Anneli Adler, Joannès Guillemot, Pedro H. S. Brancalion

**Affiliations:** ^1^ Department of Forest Sciences Luiz de Queiroz College of Agriculture, University of São Paulo Piracicaba São Paulo Brazil; ^2^ Department of Crop Production Ecology Swedish University of Agricultural Sciences Uppsala Sweden; ^3^ Department of Southern Swedish Forest Research Centre Swedish University of Agricultural Sciences Lomma Sweden; ^4^ CIRAD UMR Eco&Sols Montpellier France; ^5^ Eco&Sols Univ. Montpellier, CIRAD, INRAe, Institut Agro, IRD Montpellier France; ^6^ Center for Carbon Research in Tropical Agriculture (CCarbon) Piracicaba São Paulo Brazil; ^7^ Re.Green Rio de Janeiro Rio de Janeiro Brazil

**Keywords:** biodiversity, climate‐change adaptation, forest management, forest restoration, forestry, natural climate solutions, nature‐based solutions

## Abstract

Mixed planted forests (MPF) are increasingly recognized for their potential to reconcile forest productivity with biodiversity conservation and ecosystem services, such as climate adaptation and mitigation. They can be mixed in multiple ways, but the lack of a standardized procedure to compare mixture levels has hindered the exploration of their potential benefits, rendering the term “mixed” too generic to support meaningful comparisons across studies or informed decision‐making. This study aims to (i) conduct a global literature review of the characteristics of MPF established for production (i.e., excluding biodiversity‐oriented forests), and (ii) develop an index to classify production‐oriented planted forests according to their level of mixture. We systematically screened the literature and selected 153 studies covering 253 MPF stands, from which we extracted detailed information on management practices using 10 criteria related to species composition (e.g., number of species, genetic diversity, species origin, functional composition) and structural/spatial design (e.g., spatial distribution, age heterogeneity, understory natural regeneration). We propose an index to classify MPF according to mixture level (scaled to a 0–1 range) based on a multi‐criteria decision analysis using the PROMETHEE II outranking method. The MPF diversity index serves as a classification tool for MPF designs based on their relative dominance across multiple indicators. The results revealed that most published MPF worldwide occupy low to intermediate positions along the diversity gradient (index mean = 0.48), indicating a diversity gap even among intentionally diverse production‐oriented planted forests. The proposed index provides a transparent and comparative classification of MPF designs that can support policy and management decisions by highlighting differences in diversity‐related design attributes.

## Graphical abstract

We conducted a global review of production‐oriented mixed planted forests (MPF) and developed a standardized index to classify their admixture level using a multi‐criteria decision analysis (PROMETHEE II). The review considers 153 studies and 253 plots, and the MPF Index (scale 0–1) integrates multiple design indicators related to species composition, spatial arrangement, and silvicultural system. The results reveal that most production‐oriented MPF worldwide occupy low to intermediate positions along the diversity gradient, indicating a diversity gap even among intentional MPF. The index provides a transparent and comparative framework for assessing admixture levels and supporting evidence‐based forest management and policy decisions.

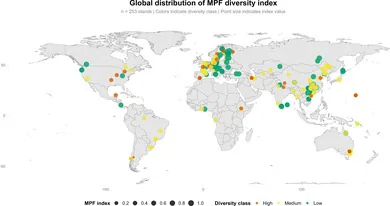

## 1 Introduction

Mixed planted forests (MPF), which combine multiple tree species within the same stand, have emerged as a promising nature‐based solution to enhance the resilience of commercial forestry systems to climate change (Pretzsch and Schütze 2016; Zeller et al. 2017), as well as to provide co‐benefits for biodiversity and ecosystem services (Pretzsch 2014). However, the capacity of MPF to enhance resilience, store carbon, conserve biodiversity, and provide myriad ecosystem services is influenced by their level of mixture (Crowther et al. 2019; Messier et al. 2022; Pan et al. 2011; Warner et al. 2023). Understanding and quantifying the level of stand mixture has become increasingly important for comparing MFP designs and exploring their potential associations with resilience and ecosystem services provision (Grossman et al. 2018; Jactel et al. 2017; Paquette and Messier 2010; Verheyen et al. 2016). Such comparisons are essential for identifying mixture designs that best achieve specific objectives under different socio‐ecological conditions. However, the lack of a standardized method for quantifying the levels of mixture of planted forests has limited reliable assessments.

MPF still represents a minimal fraction of global planted forest area (FAO and UNEP 2020), but this underrepresentation may change soon, given the increasing recognition of MPF as a valuable tool to increase forestry sustainability and resilience to climate change. With global timber demand projected to increase 50% by 2050 (FAO 2022), coupled with international commitments to restore millions of hectares of degraded forest landscapes (Brancalion and Holl 2020; Dave et al. 2019; Sewell et al. 2020), MPF represents a strategy to balance production and conservation objectives (Cerullo et al. 2024; Li et al. 2022). Then, comparing MPF mixture levels can be critical to guide decisions on the adoption of planted forest schemes in commercial forestry, based on the likelihood of generating targeted benefits in each socio‐ecological context.

The current definition of MPF as planted forests established through planting or seeding and composed of two or more tree species (FAO 2020) is often too restrictive to capture the full diversity of configurations and management approaches enabled by silvicultural practices. These systems can differ widely in multiple dimensions, including the number of species involved, their functional identity (Li et al. 2022), and their level of genetic diversity (Bauhus et al. 2017). Moreover, MPF may vary in spatial arrangement, ranging from species intermingled within the stand to structured designs, such as alternating rows or random distributions (Beugnon et al. 2025), as well as in temporal composition, depending on whether species are established simultaneously or sequentially through natural regeneration or enrichment plantings (Messier et al. 2022). Silvicultural interventions also play a central role, particularly those that promote or suppress natural regeneration in the understory—a central process to increase the ecological complexity of plantations (Simões et al. 2024).

Given this complexity, relying on a binary classification of planted forests as “mixed = ≥ 2 species” or “monoculture = 1 species” may overlook important distinctions, with marked implications for practice. For example, a stand consisting of two closely related species planted in rows and managed intensively is likely to provide a markedly different suite of ecosystem services than a structurally diverse stand with 10 species and active natural regeneration (Forrester and Pretzsch 2015; Brancalion et al. 2025). An index‐based approach allows for a more precise and standardized classification of these systems by quantifying their relative position along a gradient of mixed forest design attributes in a production context. Although several important indices have been developed to assess forestry and forest ecosystem characteristics (Acker et al. 1998; Geburek et al. 2010; Jia et al. 2023; Li, Jian, and Lu 2024; Mondal et al. 2024; Storch et al. 2018; Venema et al. 2005), none have been specifically designed to classify how mixed a planted forest is.

The objectives of this study are: (i) to conduct a comprehensive review of the global literature on MPF, encompassing the full range of configurations of mixed planted forests established for production, including both commercial and experimental systems; (ii) to develop an index to classify the level of mixture of MPF for production, based on the literature review, integrating key spatial, temporal, compositional, and management attributes.

## 2 Materials and Methods

### 2.1 Literature Review

We searched the Web of Science platform for the following keywords: “mixed tree planting”; “intercropping”; “mixed plantation”; “mixed plantations”; “mixed‐species plantations”; “mixed forest plantations”; “polyculture” written in English and selected only research articles. We got 2646 results. All paper titles and abstracts were read. We excluded papers that analyzed data of plants grown in greenhouses/pots or were not about tree species for silvicultural purposes. When it was not clear if the mixed forest was managed for production or not, we did not include the article in the review, the same for urban spaces/ornamental, silvopastoral systems and forest restoration purposes.

Restoration‐oriented plantings, conservation plantings, and biodiversity experiments without direct management application for production were excluded. Including them would introduce incomparable systems that would obscure patterns specific to production forestry. We filtered papers between 2004 and 2024 to prioritize analyzing stands that reflect recent practices in forestry over historical/old ones to better influence contemporary decisions on how production‐oriented planted forests are designed and managed. We also restricted to English‐language articles for practical feasibility and to avoid potential biases in selecting some languages, given our objective to provide a global overview on this topic. This first selection resulted in 388 papers and, as expected, excluded some influential papers on this topic that were not captured due to the search decisions explained above (Kelty 2006; Lamb et al. 2005; Parrotta 1999; Piotto et al. 2004).

Subsequently, each selected article was analyzed, and a table was filled with all relevant information available, including: the general subject of the study, location (latitude and longitude), country, continent, climate zone, growth synchrony, stand density (trees/ha), timber quality (low, medium, or high), main purposes, planting area (ha), spacing, classification as experimental or commercial designs, number of main species, proportion of each species (equal or not and details of the proportion if available), classification of the species as native, exotic, or both in the stand, genetic diversity level of the main species, type of species (coniferous, broadleaved, or both), the presence or not of nitrogen‐fixing species and understory management practices (Table 1). Studies lacking basic information about the stands were excluded. This second filtering process resulted in 153 papers, corresponding to 253 MPF stands. Some information was obtained from Supporting Information, official project websites cited in the article, and basic details about the planted species (e.g., whether they are considered native or exotic, their primary purposes) were gathered from the literature. The list containing each article included in the database is available in the Supporting Information (Table S1).

**TABLE 1:** Description of the indicators used to compose the MPF diversity index. For all indicators, higher values were preferred, meaning that higher scores indicate greater diversity or more desirable management conditions.

Category	Indicator	Description	Measurement	Range	Key references
Species composition	Number of species	Species richness is a central component of biodiversity and is frequently reported as a key design attribute in MPF. Higher species richness has been associated in literature with increased resilience to pests, diseases and climate change, and provide a wider range of ecosystem services.	Numeric—continuous	≥ 2 species	(Liu et al. 2022; Hua et al. 2022; Jactel and Brockerhoff 2007; Li, Hao, et al. 2024; Messier et al. 2022; Van Der Plas et al. 2016)
	Level of genetic diversity	Genetic diversity is crucial for the adaptability and resilience of planted forests. Higher genetic diversity has a greater capacity to adapt to environmental stresses and a lower risk of catastrophic losses.	Categoric (ordinal)	This indicator was divided into: 1 = same family and genus but different species^a^; 2 = same family but different genus; 3 = species of different families	(Perry et al. 2024)
	Presence of nitrogen‐fixing species	Nitrogen‐fixing species play a key role in improving soil fertility. By enriching the soil with nitrogen, these species benefit the growth of nearby non‐nitrogen‐fixing trees and promote ecological interactions.	Binary	This indicator was divided into: 1 = presence and 0 = absence of N‐fixing species.	(Forrester et al. 2005)
	Species origin	The origin of planted species reflects biogeographic and ecological considerations associated with local adaptation, invasion risk, and conservation value. Native species are often associated with reduced ecological risk and greater compatibility with local biodiversity.	Categoric (ordinal)	This indicator was divided into: 1 = exotic species only; 2 = a mix of native and exotic species, 3 = only native species	(Pritchard et al. 2024).
	Number of main purposes	Mixed forest stands that serve multiple purposes (timber, conservation, recreation) promote the diversity of ecosystem services and increase socio‐ecological resilience.	Numeric—continuous	1–3 purposes observed	(Gamfeldt et al. 2013; Messier et al. 2022)
	Functional composition	Mixed broadleaf and coniferous stands in boreal, temperate, and subtropical regions are frequently mentioned in literature as structurally and functionally diverse planting models.	Binary	This indicator was divided differently according to the climate zone. In the tropics: 0 = only conifers; 1 = only broadleaves or mixed broadleaf/conifer. In boreal, temperate and subtropical regions: 0 = only broadleaf or conifers; 1 = mixed broadleaf/conifer^b^	(Jactel and Brockerhoff 2007; Urgoiti et al. 2023)
Structural and spatial design	Spatial distribution	Spatial distribution refers to the arrangement of species at the stand and plays a critical role in determining interspecific interactions and ecological processes. These configurations vary in the level of structural complexity and heterogeneity they introduce to the stand.	Categoric (ordinal)	This indicator was divided into: 1 = mosaics (different species planted in patches) and edges (when there is another species planted around a monocultural stand); 2 = rows; 3 = species randomly distributed within a stand^c^	(Beugnon et al. 2025; Gregorius 1991; Pretzsch and Forrester 2017; Uprety et al. 2025)
	Proportion of each species in the stand	The relative proportion of species affects the structure and function of the ecosystem. Mixed forests with balanced proportions can promote more interactions among species, such as facilitation and complementarity, increasing productivity and stability. And indicates that there is no species that heavily dominates the stand.	Binary	This indicator was divided into: 0 = unequal proportions; 1 = equal proportion	(Feng et al. 2022; Liu et al. 2018)
	Even‐aged management	Mixed forest stands with age stratification promote greater structural heterogeneity, benefiting biodiversity and increasing resilience to disturbances. Age diversity can also improve production and ecosystem services.	Binary	This indicator was divided into: 0 = even‐aged; 1 = age‐heterogeneous	(Savilaakso et al. 2021)
	Natural regeneration in the understory	The presence of spontaneous species in the understory indicates active ecological processes and contributes to biodiversity and ecosystem resilience. Allowing natural regeneration can improve habitat structure in the long term.	Binary	This indicator was divided into: 0 = understory was suppressed, by mechanical or biological methods; 1 = spontaneous species were retained.	(Markku Kanninen 2010)

^a^ This classification reflects widely used taxonomic hierarchies as a proxy for genetic distance and allows genetic diversity to be incorporated into a comparative index without assuming linear relationships or direct functional outcomes (Hamrick et al. 1992; Porth and El‐kassaby 2014).

^b^ The functional composition criteria was applied differently across climate zones to ensure ecological relevance and avoid penalizing stands for diversification pathways that are not naturally feasible. In boreal, temperate, and subtropical regions, conifers occur naturally and abundantly, so mixed broadleaf‐conifer forests are ecologically viable and well‐documented. In these regions, we considered mixed stands as the preferred condition = 1, while stands with only one functional group = 0, reflecting a missed opportunity for functional complementarity. In the tropics, however, conifers are naturally rare, and mixed broadleaf‐conifer forests are not considered a common management. Applying the same criteria would systematically underestimate the diversity potential of tropical MPF. Therefore, in tropical stands, we considered any stand that is not “conifer‐only” (i.e., broadleaf‐only or mixed) = 1, aligning the criteria with the ecological realities of conditions while maintaining the index's core principle: rewarding attainable functional diversification.

^c^ Random: Intimate mixing, high co‐occurrence; Rows: Operational mixing, common in commercial planted forests; Edges: Limited integration; Mosaics: Deliberate spatial segregation. These categories were ordered according to the degree of within‐stand spatial integration, with random mixtures representing the highest level of spatial mixture and mosaics representing the lowest level. This classification reflects increasing levels of spatial segregation and structural differentiation and is widely used to describe planting designs in forestry, allowing consistent comparison across heterogeneous case studies and reflects the intensity of spatial mixing within the stand (Beugnon et al. 2025).

### 2.2 MPF Diversity Index

The initial candidate indicators included in the MPF index were selected based on key measurable attributes commonly recognized as relevant to characterize the structural, spatial, and compositional design of planted forests. We prioritized indicators that are easy to understand and could be consistently extracted as basic information from the reviewed articles, to provide transparency and replicability. The indicators were separated into two key categories: Species composition and structural and spatial design.

The selected indicators represent characteristics that have been widely discussed in literature as being associated with forest resilience, productivity, and the provision of ecosystem services at the stand level. Therefore, our intention is to present an index that provides a comparative synthesis of the level of mixture of MPF according to multiple indicators and describes how mixed are planted forests for production forestry.

### 2.3 Construction of the Diversity Index Using Multi‐Criteria Decision Analysis

To integrate the selected indicators into a single comparative metric, we applied a multi‐criteria decision analysis (MCDA) framework based on the PROMETHEE II outranking method (Brans and De Smet 2016). This approach allows the aggregation of heterogeneous quantitative and qualitative indicators into a single net outranking flow.

Preference functions and thresholds: Following best practices in multi‐criteria decision analysis, we applied Type I (usual) preference functions for binary criteria (proportion of each species in the stand, natural regeneration in the understory, even‐aged management, presence of nitrogen‐fixing species, and functional composition) and Type V (linear) functions for ordinal and continuous criteria (number of species, level of genetic diversity, species origin, number of main purposes, spatial distribution). Indifference thresholds (*q*) were set to 0 for all linear criteria, meaning any positive difference in the preferred direction contributes to preference. Preference thresholds (*p*) were set to the empirical range of each criterion, ensuring that a difference spanning the full observed range results in full preference.

All criteria received equal weights (1/10 each) to avoid subjective, a priori, judgments. A sensitivity analysis comparing alternative weighting schemes—(i) equal cumulative weight per category (species composition vs. structural/spatial design, each 0.5) and (ii) based on ecological aspects weights prioritizing species richness, genetic diversity, natural regeneration, and spatial distribution—confirmed the robustness of the ranking (Spearman's *ρ* ≥ 0.975). More details available in the Supporting Information.

For each stand, we calculated a positive outranking flow (φ^+^) and a negative outranking flow (φ^−^) based on pairwise comparisons across all criteria. The MPF diversity index was then defined as the net outranking flow: φ = φ^+^ − φ^−^, where higher values indicate stands that outperform others across multiple diversity dimensions.

Since the PROMETHEE index is inherently relative, we normalized the values to a 0–1 scale using min‐max normalization. To classify stands into diversity classes, we applied hierarchical clustering (Ward's method) on the (φ^+^, φ^−^) coordinates (Beugnon et al. 2025; Gregorius 1991). A three‐cluster solution (low, medium, high diversity) was retained for interpretability.

### 2.4 Identification of the Most Influent Criteria: Action Profile and GAIA Plan

To identify the most influential criteria, we computed an Action Profile (Brans and De Smet 2016), which displays the mean normalized value of each indicator per diversity class. Criteria with the largest differences between high and low classes were considered the most discriminating. To complement this, we computed a GAIA plan (Brans and De Smet 2016), a principal component analysis (PCA) that projects stands and criteria onto a two‐dimensional plane, where longer vectors indicate stronger influence on the overall ranking. Together, these analyses provide complementary insights: the Action Profile reveals which criteria most differentiate the diversity classes, while the GAIA plan identifies which criteria most influence the overall ranking.

## 3 Results

### 3.1 Literature Review

**FIGURE 1:**
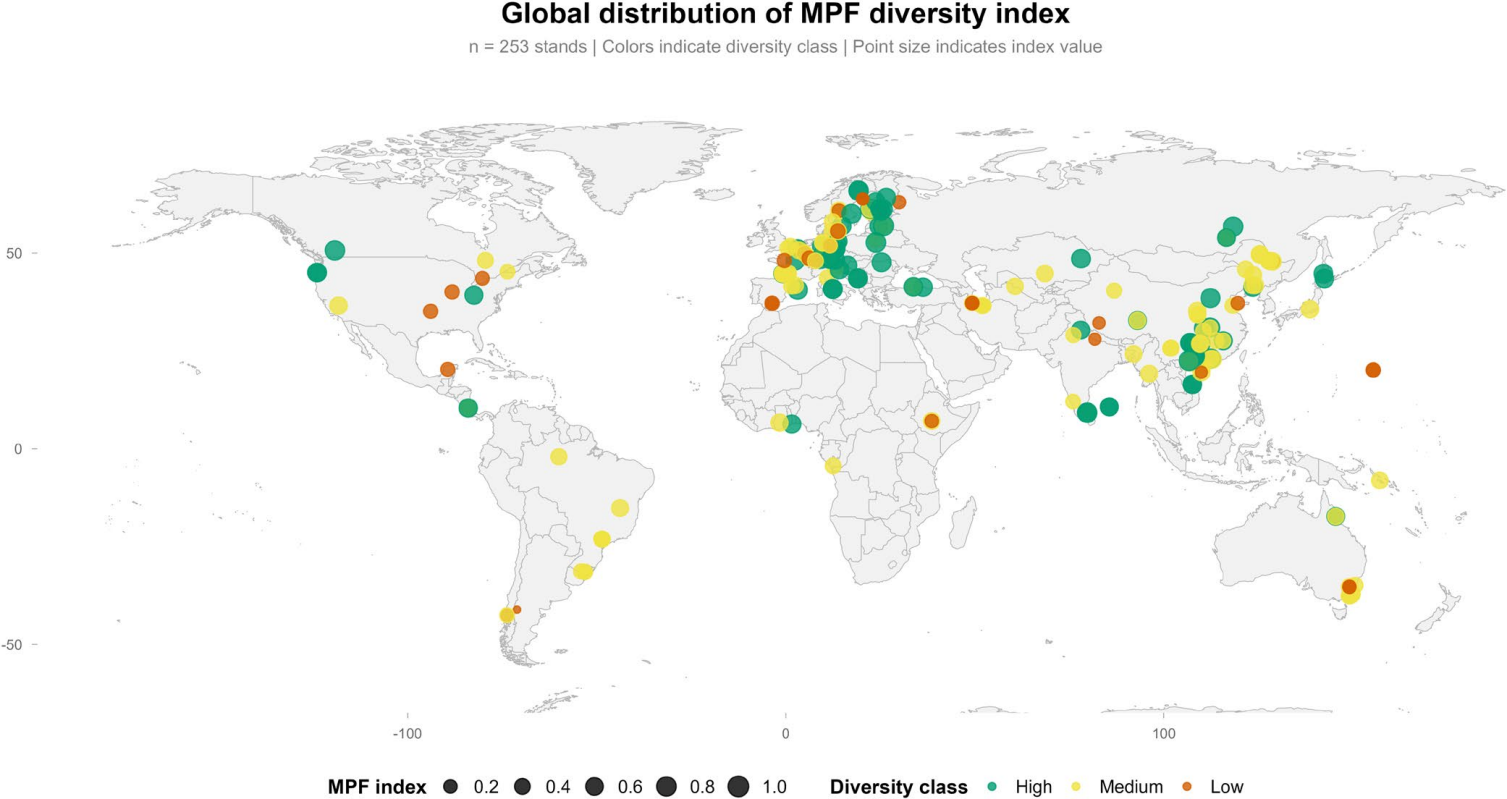
Global distribution of studies on mixed stands, with circle size representing the MPF index score and color indicating diversity class (high, medium, or low) based on the index. Map lines delineate study areas and do not necessarily depict accepted national boundaries.

**FIGURE 2:**
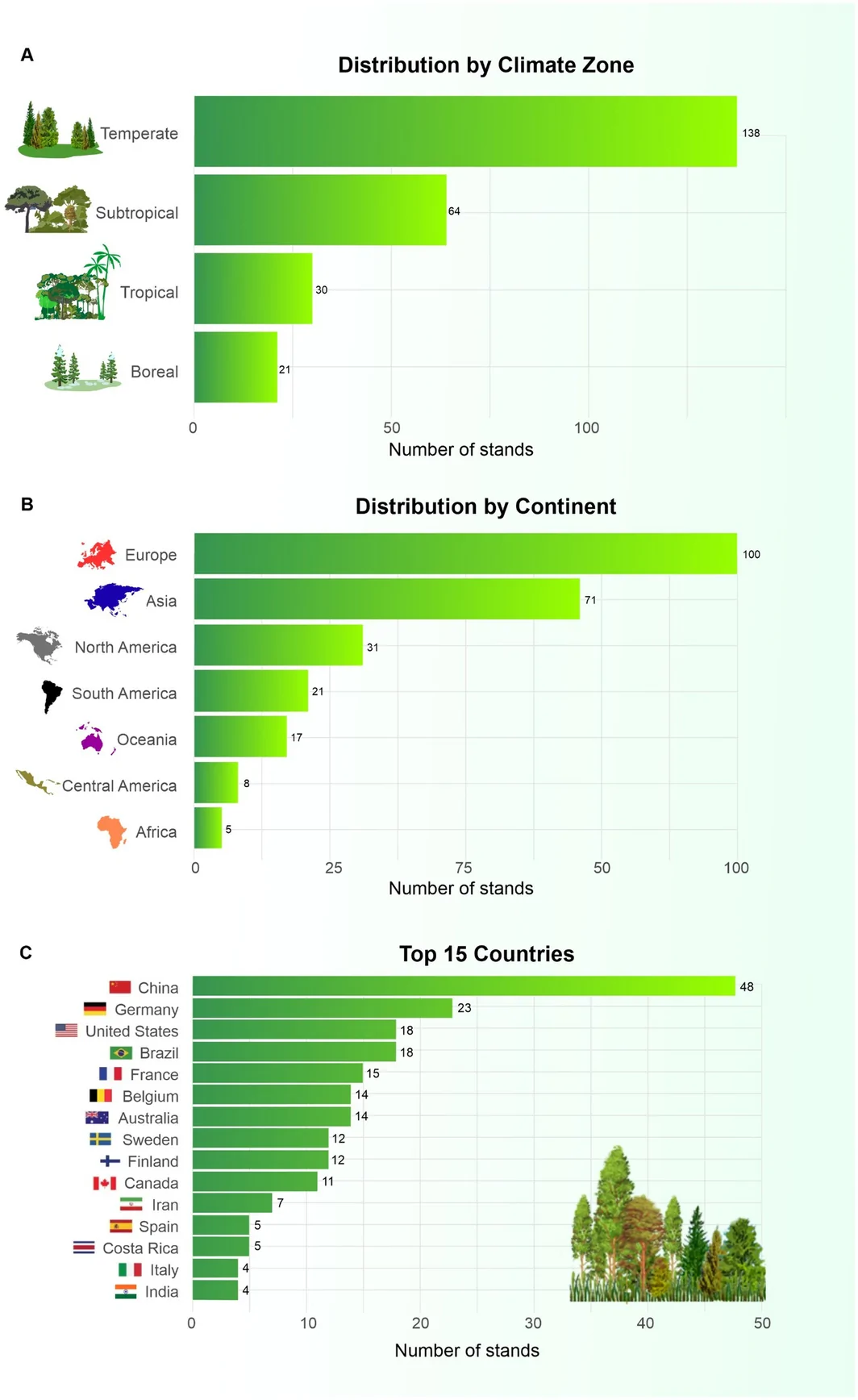
Distribution of studies on mixed stands according to the most frequent continents (A), countries (B), and climate zones (C).

Europe stood out as the continent with the highest number of studies on MPF (Figure 1), particularly those composed of combinations of broadleaves and conifers in the western region, followed by Asia, where most stands are in China. In contrast, there was a scarcity of studies addressing mixed stands in the African continent and in the boreal regions of the globe (Figures 2A and S1 in Supporting Information). Despite the expectation of a greater focus on tropical regions, due to their higher biodiversity and forest biomass, the studies were predominantly conducted in temperate climatic zones (Figure 2B). Among countries, China had the highest number of studies on mixed stands, followed by Germany, United States of America, and Brazil (Figure 2C).

The most frequent species reported in the evaluated studies on MPF were *Betula pendula, Picea abies*, and *Pinus sylvestris* , whereas the most frequent genera were *Pinus*, *Betula, Quercus*, and *Eucalyptus*. Among the 253 stands analyzed, 216 (85.4%) were classified as experimental designs and 37 (14.6%) as commercial designs.

### 3.2 Distribution of the MPF Index Based on PROMETHEE

**FIGURE 3:**
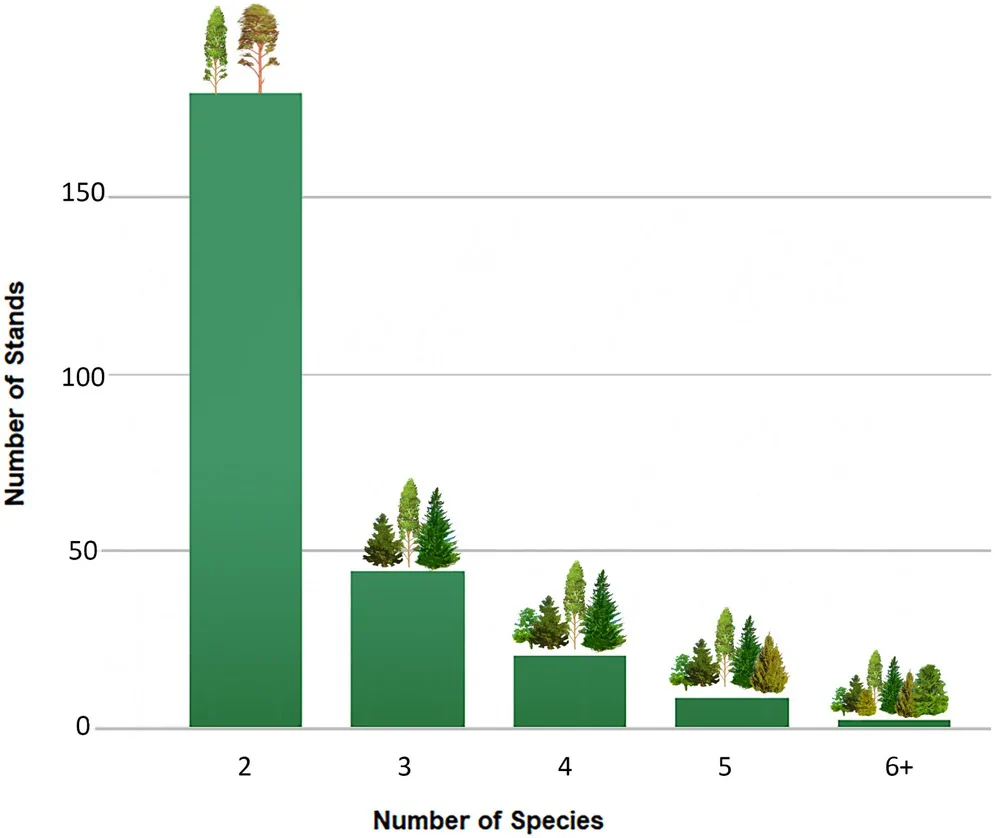
Number of species cultivated in the studies on mixed stands for production from the literature review.

The MPF production‐oriented stands usually include only two species (Figure 3), comprising 67% of the total, while stands with three or more species are less common (Figure 3). This shows a dominance of low species richness in MPF, at least when compared to native forests. Only 12% employed a management approach that retained spontaneous understory species (i.e., natural regeneration was not systematically suppressed through mechanical or chemical weeding).

**FIGURE 4:**
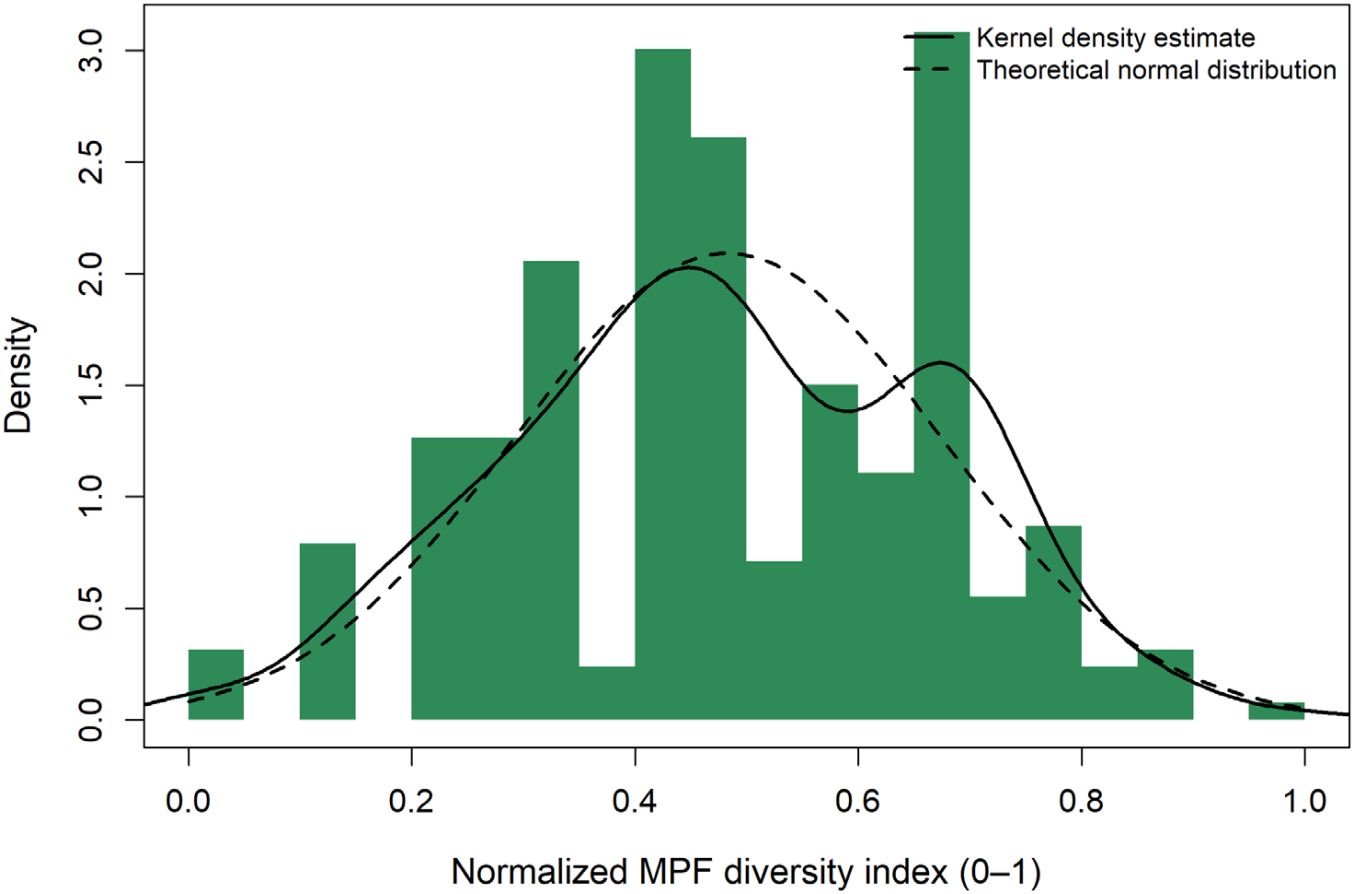
Distribution of the normalized PROMETHEE‐based MPF index (0–1) across the analyzed MPF stands (*n* = 253). Bars represent density distribution. The solid line indicates the kernel density estimate of the observed values, and the dashed line represents the theoretical normal distribution based on the sample mean and standard deviation.

The PROMETHEE II multi‐criteria analysis produced a continuous net outranking flow (φ) for each stand, ranging from −0.3502 to 0.3748. After min‐max normalization to a 0–1 scale, the MPF diversity index ranged from 0 to 1, with a mean of 0.4830 and a median of 0.4828 (SD = 0.1908). The distribution was approximately symmetric (skewness = 0.054; Figure 4).

### 3.3 Hierarchical Clustering Classification

We applied hierarchical clustering on the (φ^+^, φ^−^) coordinates. Using Euclidean distance and Ward's method, we identified three stable clusters (silhouette width = 0.45 for *k* = 3):

Cluster 1 (high diversity, *n* = 60, 23.7%): Stands with the highest index values (mean normalized index = 0.731). These stands excelled in age heterogeneity (0.92), spatial distribution (0.87), functional composition (0.97), species origin (0.94), and permission of natural regeneration (1.00). Notably, species richness remained modest (mean = 2.63 species), indicating that high MPF index scores can be achieved through multiple diversity dimensions.

Cluster 2 (medium diversity, *n* = 118, 46.6%): Stands with intermediate index values (mean = 0.501). This cluster showed moderate values across most indicators, with higher scores for genetic diversity (0.94) and stand proportional evenness (0.56), but lower values for spatial distribution (0.16) and age heterogeneity (0.25). The medium‐diversity class represents the largest portion of analyzed stands.

Cluster 3 (low diversity, *n* = 75, 29.6%): Stands with the lowest index values (mean = 0.257). These stands showed consistently low values across nearly all indicators, including functional composition (0.13), spatial distribution (0.00), age heterogeneity (0.01), and natural regeneration (0.23). Species richness was also lowest in this class (mean = 2.24 species).

The Spearman correlation between species richness and the MPF index was *ρ* = 0.245 (*p* < 0.001). While statistically significant, this weak correlation confirms that the MPF index captures multiple dimensions of diversity beyond simple species counts. Stands with the same number of species varied widely in their index values, reflecting differences in genetic diversity, native species status, functional composition, understory management, spatial arrangement, and other criteria.

### 3.4 Action Profile of Diversity Classes

To identify which criteria most strongly discriminate between diversity classes, we computed an Action Profile, showing the mean normalized value of each indicator per cluster (Table 2) (Brans and De Smet 2016). The high diversity class was characterized by notably higher values for natural regeneration (1.00 vs. 0.23 in low), functional composition (0.97 vs. 0.13), species origin (0.94 vs. 0.47), age heterogeneity (0.92 vs. 0.01), and spatial distribution (0.87 vs. 0.00). These five criteria represent the most influential dimensions for achieving high MPF diversity scores. Conversely, the low diversity class showed consistently low values across most indicators, particularly for spatial distribution, age heterogeneity, functional composition, and understory natural regeneration, indicating that these stands fail to integrate multiple diversity dimensions simultaneously.

**TABLE 2:** Action profile—mean normalized values per diversity class.

Indicator	High (*n* = 60)	Medium (*n* = 118)	Low (*n* = 75)
Age heterogeneity	0.92	0.25	0.01
Number of purposes	0.12	0.21	0.17
Spatial distribution	0.87	0.16	0.00
Number of species	0.16	0.13	0.06
Proportion of each species	0.18	0.56	0.63
Species origin	0.94	0.78	0.47
Genetic diversity	1.00	0.94	0.79
Functional composition	0.97	0.54	0.13
Presence of N‐fixing species	0.15	0.37	0.37
Understory natural regeneration	1.00	0.69	0.23

**FIGURE 5:**
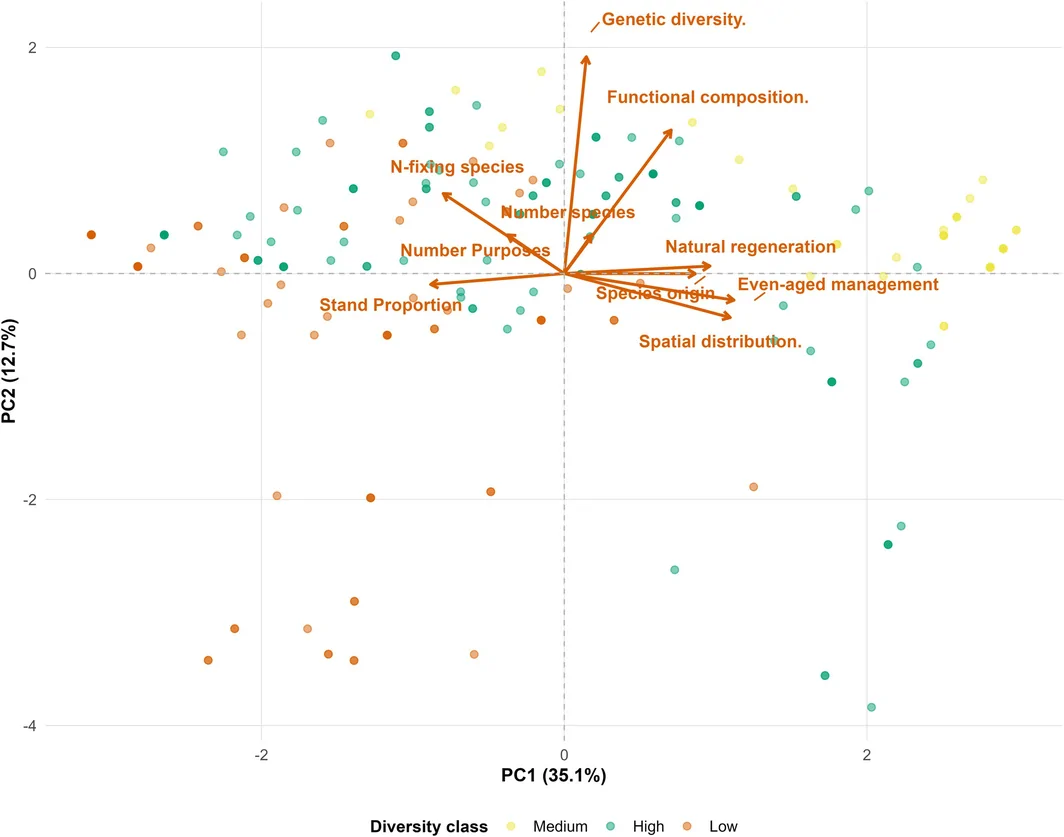
The GAIA plan shows the projection of stands (points) and criteria (arrows) onto the first two principal components. Points are colored by diversity class (high, medium, and low). The length of each arrow indicates the contribution of the corresponding criteria to the overall ranking.

To complement the Action Profile and identify the criteria that most influence the overall ranking, we computed a GAIA plan on the normalized indicator matrix (Brans and De Smet 2016). The first two principal components from the principal component analysis (PCA) explained 47.8% of the total variance (PC1: 35.1%, PC2: 12.7%). The GAIA plan revealed that the criteria contributing most to the overall ranking were genetic diversity, broadleaf/conifer mixing, and N‐fixing species. Together, these analyses indicate that both compositional attributes and structural attributes are influential dimensions for achieving high MPF diversity scores (Figure 5).

### 3.5 Global Average Profile—Radar Plot

**FIGURE 6:**
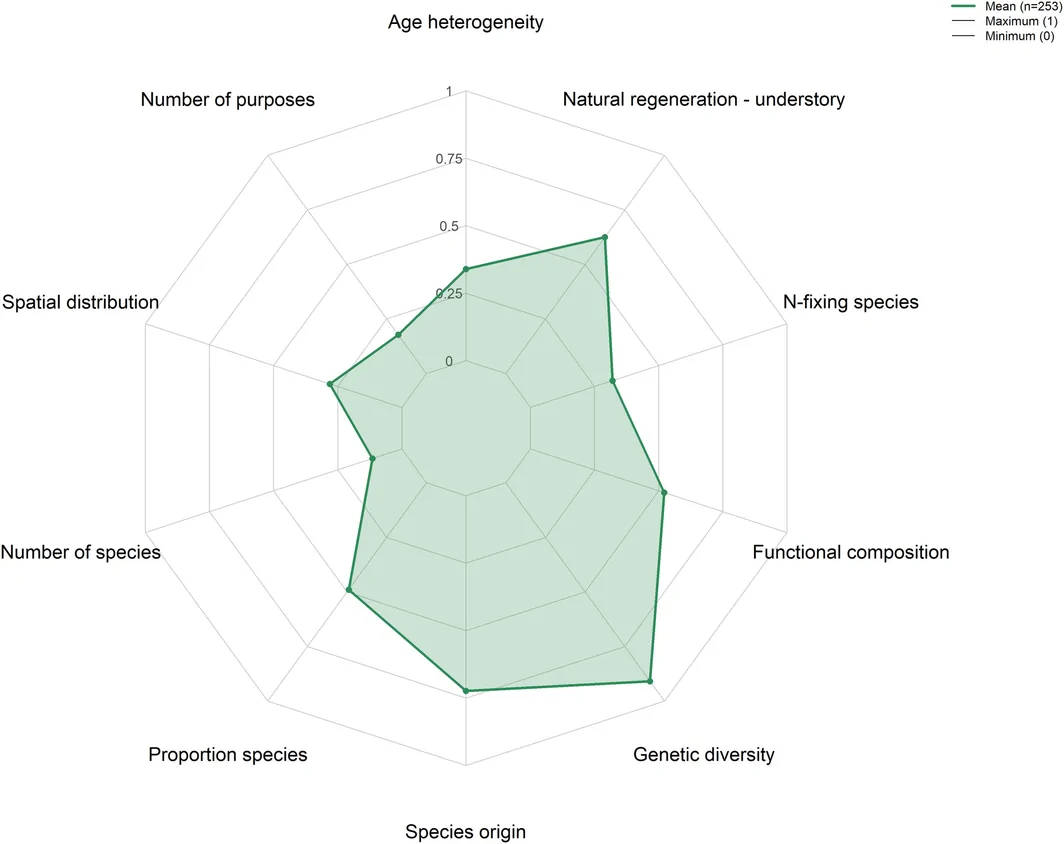
Radar plot illustrating the global average profile of diversity‐related design indicators across 253 studies of MPF for silviculture worldwide. Values are relative to the empirical range observed in the dataset and illustrate the global profile of MPF design attributes.

The radar plot (Figure 6) illustrates the global average profile of diversity‐related design attributes across the 253 analyzed MPF stands. Values closer to 1 indicate that, on average, stands are closer to the highest observed level of that attribute within the dataset.

Genetic diversity showed the highest global average (0.91), followed by species origin (0.72) and natural regeneration (0.63). Functional composition and proportional evenness showed intermediate values (0.52 and 0.49, respectively). Even‐aged management showed a global average of 0.34, indicating that 66% of MPF stands are even‐aged (i.e., all trees in the forest stand were established simultaneously), while 34% are age‐heterogeneous. This finding is consistent with current practices in forestry, where even‐aged management predominates.

The lowest global averages were observed for number of species (0.11), number of purposes (0.18), and spatial distribution (0.28), suggesting that these are the least represented diversity dimensions across the reviewed MPF stands.

## 4 Discussion

Although MPF adoption may represent an important advance compared to the dominance of tree monocultures in commercial forestry, especially when monoclonal plantations are employed, there remain significant opportunities to enhance stands' resilience and environmental contribution through diversification (Liu et al. 2018; Wang et al. 2022). The MPF index based on the PROMETHEE method developed in this study provides an accessible and replicable framework for comparatively classifying the level of mixture of MPF stands in different socio‐ecological contexts, going beyond the mere number of species to include broader ecological and management aspects by synthesizing multiple mixture‐related design attributes that have been widely discussed in the literature as relevant to the potential performance of MPF for multifunctionality (Li et al. 2022).

Despite increasing global interest in MPF, our results show that most stands are concentrated at low to intermediate values along the PROMETHEE‐based index. More than half of the analyzed MPF were outranked by most other systems across the selected indicators. This reveals a relatively low level of compositional and structural mixture, even among stands nominally classified as “mixed”. This indicates that, in practice, most production‐oriented MPF remain closer to simplified monocultures rather than representing heterogeneous planted forest designs integrating diversity‐oriented features.

Compared to other parts of the globe, MPF stands are more thoroughly scientifically documented in Europe, where they are predominantly established in temperate regions (Bauhus et al. 2017). In contrast, tropical regions, despite their remarkable biodiversity, are still largely dominated by extensive monocultures, often composed of a single clone (Iezzi et al. 2018), with limited implementation of MPF in commercial stands, although many experiments with mixtures have been empirically tested (Brancalion et al. 2020; Piotto et al. 2004). This widespread reliance on simplified commercial planted forests in the tropics represents a missed opportunity to harness and promote the natural ecological potential of these regions (Cagnoni et al. 2023). It also reinforces the utilitarian perspective of a planted forest as a mere source of raw materials, regardless of its societal and environmental values (Chazdon et al. 2016). The strong separation between commercial forestry and species diversity limits the ability of tropical MPF to provide the ecological and socio‐economic benefits that more diverse and integrated systems could deliver (Erskine et al. 2006).

The Radar Plot, Action Profile, and GAIA Plan provided here convergent insights into the dimensions of diversity that matter most in MPF. The Action Profile further identified natural regeneration, functional composition, species origin, and age heterogeneity as the most discriminating criteria between diversity classes, while the GAIA Plan revealed that genetic diversity, functional composition, and N‐fixing species were the most influential indicators considering the overall MPF diversity index. This multi‐dimensional perspective aligns with the emerging paradigm that forest multifunctionality depends on multiple facets of biodiversity (Gamfeldt et al. 2013; Messier et al. 2022; Van Der Plas et al. 2016), and suggests that silvicultural practices promoting native species, retaining natural regeneration, mixing functional groups, and creating structural complexity can effectively enhance MPF diversity.

However, an important trade‐off must be acknowledged. While higher mixture scores reflect ecologically richer systems, they may also imply greater complexity in terms of planning, management, and operational costs (Summers et al. 2015). Diversified MPF often requires more technical knowledge, infrastructure, and long‐term investment, which may result in the implementation of complexity and higher costs, serving as key barriers based on the perspectives of decision‐makers (Bulascoschi Cagnoni et al. 2026). The main challenge is being able to maximize ecological value and maintain practical viability, especially for landowners and companies that prioritize operational simplicity and effectiveness.

The proposed index can support multiple policy and management applications by providing a transparent and comparative classification of production‐oriented MPF designs. This can be used as a decision‐support tool supporting the prioritization of plantation designs that incorporate a broader range of mixture‐related attributes commonly associated in the literature with resilience and ecosystem service potential, going beyond the simplistic evaluation of the number of species alone (Tedesco et al. 2023). Subsidies, carbon and biodiversity credits (Assmuth et al. 2024; Clifton and Mánez 2025; Croci et al. 2025; Crossman et al. 2011), and other schemes of payments for ecosystem services may be needed to improve the financial viability of MPF for production (Griscom et al. 2017; Pagiola et al. 2005), thus providing financial rewards for the broader societal and ecological benefits they provide and offsetting the potentially higher production costs of timber and non‐timber forest products (Sinacore et al. 2023). Our index may contribute to these initiatives by providing a robust and transparent framework for quantifying the expected contribution of different mixed stands designs—relative to the context in which they are employed—to biodiversity provision, thereby supporting the design of payment schemes that better reflect their overarching contributions to society. This potential is underscored by recent findings from Norway and Sweden showing that citizens are willing to contribute approximately €10–14 per month via tax payment to support biodiversity‐enhancing management of family‐owned production forests, indicating a promising societal basis for payment schemes that reward forest management practices delivering public environmental benefits (Kim et al. 2024).

Forest certification programs, such as the FSC (Forest Stewardship Council) and PEFC (Programme for the Endorsement of Forest Certification), represent promising mechanisms for implementing the MPF index on a broader scale. These organizations value biodiversity and already incorporate principles of biological diversity conservation and ecosystem services procedures (FSC Principle 6; PEFC Criterion 6) (Forest Stewardship Council 2025). The application of our index could help differentiate planted forest designs based on their species composition and structural/spatial attributes, potentially generating added value for products originating from more diverse systems and aligning economic and environmental interests.

It is important to highlight, however, that the implementation of a given MPF, even when a more mixed method is used, is not a guarantee of enhanced environmental outcomes. There are multiple other factors influencing their outcomes, such as the choice of species based on their complementarity, previous land use, landscape attributes, soil and climate, and disturbances, so monitoring and adaptive management remain crucial (Forrester and Bauhus 2016). Recognizing and promoting more mixed planted forests can be a first step towards achieving climate and sustainability goals, balancing forest production and conservation. We believe that promoting mixture in production‐oriented designs, enhancing the accuracy and transparency of how these systems are classified and communicated to stakeholders and society through the proposed index, can markedly contribute to this transformation.

A limitation of our index is that one axis of functional diversity not considered by our index is leaf phenology (deciduous vs. evergreen). Although partially correlated with the distinction between broadleaf and coniferous trees, leaf phenology influences seasonal patterns of light availability, litter quality, and decomposition rates (Cornwell et al. 2008), understory microclimate, and habitat provision for fauna (James and Wamer 1982). These factors may be particularly relevant in temperate and boreal regions, where deciduous and evergreen mixtures are associated with increased resilience and habitat heterogeneity. Another challenge that remains for future research is understanding how the weights of the criteria should relate to specific ecosystem services. Different services may be associated differently with mixing attributes—carbon storage with species richness, habitat provision with native species, and understory regeneration. Future studies could investigate these empirical relationships, potentially providing information for context‐specific refinements.

## Author Contributions

**Leticia Bulascoschi Cagnoni:** conceptualization, investigation, writing – original draft, methodology, validation, formal analysis, writing – review and editing, visualization. **Martin Weih:** conceptualization, writing – review and editing, funding acquisition, supervision. **Anneli Adler:** conceptualization, writing – review and editing, supervision. **Joannès Guillemot:** conceptualization, writing – review and editing, methodology, investigation. **Pedro H. S. Brancalion:** supervision, writing – review and editing, conceptualization, investigation, funding acquisition.

## Funding

This research was funded through the 2019–2020 BiodivERsA joint call for research proposals, under the BiodivClim ERA‐Net COFUND program (MixForChange project), with funding from FAPESP (processes numbers: 2019/24318–6 and 2021/14062–4) and the Swedish Research Council FORMAS (project number 2020–02339).

## Conflicts of Interest

Pedro H.S. Brancalion is a partner at Re.green, a restoration company. The other authors declare no conflicts of interest.

## Supporting information


**Data S1:** gcb71111‐sup‐0001‐Supinfo.docx.
**Table S1:** Summary of papers included in the review—each paper can contain more than one stand.
**Figure S1:** MPF diversity index by continent. Color circles according to the diversity class (high, medium and low).

## Data Availability

The data that support the findings of this study are openly available in https://zenodo.org/records/21709121.
