# Supplementary material for: How Mixed Are Production‐Oriented Planted Forests? A Global Review and Classification Approach
Source: Glob Chang Biol. 2026 Sep 18;32(9):e71111. doi: 10.1111/gcb.71111 (PMC13589153; doi:10.1111/gcb.71111)
Supplement: Supplementary file 1 [file GCB-32-e71111-s001.docx]

**Supplementary Material**

Table S1: Summary of papers included in the review – Each paper can contain more than one stand

| **ID Paper** | **Paper references** | **Stand location (Country)** | **Main General Paper Topic** |
| --- | --- | --- | --- |
| 1 | (Hirai et al. 2005) | Brazil | Soil physics |
| 2 | (Király and Ódor 2010) | Hungary | Epiphytic bryophytes |
| 3 | (B. Zhang et al. 2024) | China | Tree water absorption |
| 4 | (Gosselin et al. 2017) | France | Bryophytic diversity |
| 5 | (Farinha et al. 2024) | France | Biotic resistance - seed bug resistance |
| 6 | (Van de Peer et al. 2016; Hicks et al. 2018; Van de Peer et al. 2018; Zhang et al. 2022) | Belgium | Soil microbial activity, biomass and community composition |
| 7 | (Z. Li et al. 2021) | Germany | Root-derived carbon via fungi and bacteria |
| 8 | (De Araujo Pereira et al. 2017; Germon et al. 2018; Waithaisong et al. 2020) | Brazil | P nutrient and soil conditions; microbiology |
| 9 | (Bai et al. 2022) | China | Litter and metallic elements |
| 10 | (Hytönen et al. 2014) | Finland | Development of B*etula spp.* in mix and pure |
| 11 | (Singhal et al. 2021) | India | Litter and decomposition |
| 12 | (Chifflot et al. 2009) | Canada | Mycorrhizal |
| 13 | (Lin et al. 2024) | China | Thinning intensity |
| 14 | (Xu et al. 2022; Li et al. 2023) | China | Eucalyptus with native species – soil microbiology |
| 15 | (Bolte et al. 2004) | Germany | Root biomass |
| 16 | (Viera et al. 2015) | Brazil | Root biomass |
| 17 | (Wolz et al. 2018) | United States of America | Conversion agriculture to mixed fruit and trees |
| 18 | (Alma et al. 2024) | Argentina | Testing ants control |
| 19 | (Clinch et al. 2009) | Canada | Testing intercropping system |
| 20 | (Rugani et al. 2015) | Belgium | Environmental impacts of mix |
| 21 | (Pech-Hoil et al. 2017) | Mexico | Mating performance of a specie |
| 22 | (Yang et al. 2010) | China | Exudates on development |
| 23 | (Forrester et al. 2005) | Australia | Stand management |
| 24 | (Bristow et al. 2006) | Australia | Mixture development |
| 25 | (Anning and McCarthy 2013) | United States of America | Fire management |
| 26 | (Hasegawa et al. 2014) | Japan | Arthropods |
| 27 | (Gilbert et al. 2013) | Finland | Rats control |
| 28 | (Redondo-Brenes and Montagnini 2006) | Costa Rica | Forest/species development |
| 29 | (Ghaley and Porter 2014) | Denmark | Agroforestry systems |
| 30 | (Akashi et al. 2021) | Japan | Forest management |
| 31 | (Rouhi-Moghaddam et al. 2008) | Iran | Forest management |
| 32 | (Li et al. 2022; Jiang et al. 2023) | China | Soil nutrients |
| 33 | (Zhao and Kane 2012) | United States of America | Forest management |
| 34 | (Zhang et al. 2017) | United States of America | Soil aspects |
| 35 | (Plath et al. 2011) | Panama | Insect herbivory |
| 36 | (Liziniewicz et al. 2016) | Poland | Forest management |
| 37 | (Harper et al. 2023) | Canada | Forest management |
| 38 | (Oliveira et al. 2024) | Brazil | forest management |
| 39 | (Stille et al. 2011) | India | Economics |
| 40 | (Perot et al. 2017) | France | Abiotic aspects |
| 41 | (Šálek and Sloup 2012) | Vietnam | Financial |
| 42 | (Vehviläinen and Koricheva 2006) | Finland | Mammals’ herbivory |
| 43 | (Formaglio et al. 2023) | Brazil | Soil nutrients |
| 44 | (Sayyad et al. 2006) | Iran | Forest/species development |
| 45 | (Soares et al. 2018; Soares et al. 2021) | Brazil | Forest/species development |
| 46 | (Leuschner 2009; Jacob et al. 2010; Jacob and Leuschner 2015) | Germany | Forest/species development |
| 47 | (H. Zhang et al. 2024) | China | Soil nutrients |
| 48 | (Männistö et al. 2024) | Finland | Natural regeneration |
| 49 | (Brockwell et al. 2011) | Australia | Inoculation bacteria |
| 50 | (Potvin and Gotelli 2008; Potvin and Dutilleul 2009; Kunert and Mercado Cárdenas 2015) | Panama | Competition |
| 51 | (Oak et al. 2010) | United States of America | Forest/species development |
| 52 | (Juchheim et al. 2020) | Germany | Forest/species development |
| 53 | (Hedwall et al. 2019) | Sweden |  |
| 54 | (Potvin and Gotelli 2008; Reverchon et al. 2015) | Salomon Island | Nutrients |
| 55 | (Rumberger et al. 2004) | Germany | Mycorrhizal |
| 56 | (Mathiasen 2009) | United States of America | Pests |
| 57 | (Fahlvik et al. 2011) | Sweden | Forest/species development |
| 58 | (Dodet et al. 2011) | France | Stand management |
| 59 | (Nelson et al. 2014) | United States of America | Stand management |
| 60 | (Hou et al. 2018) | China | Fertilization |
| 61 | (Ji et al. 2023) | China | Competition |
| 62 | (Kilpeläinen et al. 2011) | Finland | Stand management |
| 63 | (Coelho et al. 2007) | Brazil | Forest/species development |
| 64 | (Calder and St. Clair 2012; Rhodes et al. 2016; Lee Molinari et al. 2022) | United States of America | Competition |
| 65 | (Liu et al. 2018) | China | Soil biomass |
| 66 | (Puriņa et al. 2016) | Latvia | Forest/species development |
| 67 | (Steinbauer et al. 2006) | Australia | Pest management |
| 68 | (Li et al. 2016) | China | Understory management |
| 69 | (Lundqvist et al. 2013) | Sweden | Stand management |
| 70 | (Yang and Chen 2023) | China | Herbivory |
| 71 | (Palandrani and Alberti 2020) | Italy | Soil nutrients |
| 72 | (Kembel and Dale 2006) | Canada | Understory management |
| 73 | (Jucker et al. 2014) | Spain | Competition |
| 74 | (van Halder et al. 2019) | Spain | Parasites |
| 75 | (Lu et al. 2020) | China | Soil nutrients |
| 76 | (Randriamananjara et al. 2023) | Canada | Understory management |
| 77 | (Ara et al. 2024) | Canada | Understory management |
| 78 | (Lei et al. 2024) | China | Soil biomass |
| 79 | (Chen et al. 2024) | China | Competition |
| 80 | (Danilović et al. 2015) | Srpska Republic | Stand management |
| 81 | (Clark et al. 2008) | England | Forest/species development |
| 82 | (Akkemik et al. 2021) | Turkey | Plant morphology |
| 83 | (Liu et al. 2024) | China | Forest/species development |
| 84 | (Khamzina et al. 2009) | Uzbekistan | Soil nutrients |
| 85 | (Behera et al. 2018) | India | Agroforestry systems |
| 86 | (Berger et al. 2004; Fleischer et al. 2006) | Austria | Soil nutrients |
| 87 | (Niccoli et al. 2021) | Italy | Forest/species development |
| 88 | (Tang et al. 2024) | China | Hydrologics |
| 89 | (Koupar et al. 2011) | Iran | Forest/species development |
| 90 | (Kaitaniemi et al. 2007) | Finland | Parasites |
| 91 | (Bu et al. 2019) | China | Soil nutrients |
| 92 | (Danilović et al. 2015) | Hawaii | Competition |
| 93 | (Zalloni et al. 2019) | Italy | Forest/species development |
| 94 | (Akkemik et al. 2021) | Mexico | Agroforestry systems |
| 95 | (Newton and Cole 2006) | United States of America | Competition |
| 96 | (Y. Li et al. 2021) | China | Stand management |
| 97 | (Obando and Moya 2013) | Costa Rica | Wood quality |
| 98 | (Willis and Blazier 2022) | United States of America | Competition |
| 99 | (Bellone et al. 2021) | Sweden | Parasites |
| 100 | (Ding et al. 2017) | Germany | Forest/species development |
| 101 | (Castagneyrol et al. 2020; Fanin et al. 2022) | France | Soil nutrients |
| 102 | (Simončič et al. 2019) | Slovenia | Herbivory |
| 103 | (Jäkel and Roth 2004) | Germany | Parasites |
| 104 | (Wang et al. 2020) | China | Soil biomass |
| 105 | (Westaway et al. 2018) | England | Forest/species development |
| 106 | (Kaba et al. 2019) | Ghana | Soil nutrients |
| 107 | (Zhang et al. 2022; Wang et al. 2024) | Belgium | Microclimate |
| 108 | (Forrester et al. 2007) | Australia | Forest/species development |
| 109 | (Kara and Keleş 2023) | Turkey | Forest/species development |
| 110 | (Alesso et al. 2021) | Spain | Forest/species development |
| 111 | (del Río et al. 2014; Y. Li et al. 2021) | Germany | Hydrologic aspects |
| 112 | (Field et al. 2020) | France | Herbivory |
| 113 | (Loewe-Muñoz et al. 2020) | Chile | Forest/species development |
| 114 | (Valor et al. 2017) | Spain | Fire management |
| 115 | (Huber et al. 2016) | Germany | Agroforestry systems |
| 116 | (Field et al. 2019) | England | Herbivory |
| 117 | (Krišāns et al. 2022) | Latvia | Stand management |
| 118 | (Huang et al. 2017; Xu et al. 2024) | China | Soil nutrients |
| 119 | (Hombegowda et al. 2020) | India | Agroforestry systems |
| 120 | (Yan et al. 2024) | China | Soil nutrients |
| 121 | (Huuskonen et al. 2023) | Finland | Forest/species development |
| 122 | (Collet et al. 2014; Fruleux et al. 2020) | France | Hydrologic |
| 123 | (Vehviläinen et al. 2008) | Sweden | Herbivory |
| 124 | (Lagache et al. 2014) | France | Reproductive species aspects |
| 125 | (Pagano et al. 2008) | Brazil | Forest/species development |
| 126 | (Grotta et al. 2004) | United States of America | Competition |
| 127 | (Kriegel et al. 2021; Schuldt et al. 2022) | Germany | Biodiversity |
| 128 | (Lintunen et al. 2011) | Finland | Forest/species development |
| 129 | (Hurteau et al. 2007) | United States of America | Forest/species development |
| 130 | (Pretzsch and Schütze 2005) | Germany | Forest/species development |
| 131 | (L. Wang et al. 2022) | China | Carbon stocks |
| 132 | (Benomar et al. 2013) | Canada | Forest/species development |
| 133 | (Kroon et al. 2019) | Sweden | Economics |
| 134 | (Burner et al. 2014) | United States of America | Plant phenology |
| 135 | (Drössler et al. 2015) | Sweden | Forest/species development |
| 136 | (Qu et al. 2022) | China | Stand management |
| 137 | (Lang et al. 2014) | China | Competition |
| 138 | (Wambsganss et al. 2021) | Poland | Soil biomass |
| 139 | (Lemma 2012) | Ethiopia | Soil nutrients |
| 140 | (H. Wang et al. 2022) | China | Carbon stocks |
| 141 | (Zou et al. 2024) | China | Competition |
| 142 | (Isaac et al. 2007) | Ghana | Competition |
| 143 | (da Silva et al. 2009) | Brazil | Soil biomass |
| 144 | (Wang et al. 2010) | China | Soil biomass |
| 145 | (Rötzer et al. 2017) | Germany | Hydrologic |
| 146 | (Lu et al. 2024) | China | Hydrologic |
| 147 | (Schweier et al. 2019) | Germany | Stand management |
| 148 | (Cruickshank et al. 2018) | Canada | Stand management |
| 149 | (Sanaei et al. 2023) | Germany | Stand management |

**Sensitivity analysis of weighting schemes**

To test the robustness of our index to subjective weighting decisions, we compared three weighting schemes using the same preference functions and thresholds:

Equal per criterion (default): All 11 indicators received weight = 1/11 ≈ 0.0909.

Equal per category: Species composition indicators (n=5) received cumulative weight 0.5, and structural/spatial indicators (n=6) received cumulative weight 0.5, with equal distribution within each category.

Expert-informed: Higher weights were assigned to genetic diversity (0.15), natural regeneration (0.12), spatial distribution (0.12), species richness (0.10), and native species (0.10), with remaining weight distributed equally.

The sensitivity analysis (using the revised preference functions) resulted in: Equal per criterion (original) vs Equal per Category: Spearman's ρ=0.994 and Classification concordance: 93.3%. Equal vs Ecological: Spearman's ρ=0.997 and Classification concordance: 99.2%. Category vs Ecological: Spearman's ρ= 0.995.

These results demonstrate that the index is highly robust to reasonable variations in weighting (ρ ≥ 0.975).

Preference thresholds

Indifference thresholds (q) were set to 0 for all linear criteria, meaning any positive difference contributes to preference. Preference thresholds (p) were set to the empirical range of each criterion.

Figure S1: MPF diversity index by continent. Color circles according to the diversity class (high, medium and low).

**References**

Akashi, N., N. Nitta, and Y. Ohno. 2021. Effect of forest management on understory vascular plants in planted Abies sachalinensis forests. *Forest Ecology and Management* 497. Elsevier B.V.: 119521. doi:10.1016/j.foreco.2021.119521.

Akkemik, Ü., S. Genç, O. Y. Yilmaz, E. Selvi, H. Yilmaz, E. Sevgi, O. Sevgi, and F. Akarsu. 2021. Effects of growing site parameters on vessel elements of quercus ilex through turkey and evaluating in respect of forestry. *Turkish Journal of Agriculture and Forestry* 45: 599–616. doi:10.3906/TAR-2011-65.

Alesso, S. P., R. Tapias, J. Alaejos, and M. Fernández. 2021. Biomass yield and economic, energy and carbon balances of ulmus pumila l., robinia pseudoacacia L. and populus × euroamericana (dode) guinier short-rotation coppices on degraded lands under mediterranean climate. *Forests* 12. doi:10.3390/f12101337.

Alma, A. M., P. Fernández, M. N. Serra, J. Villacide, and M. Buteler. 2024. Damage reduction of Acromyrmex lobicornis in Pinus ponderosa using mixed pine species in combination with push–pull tools. *Agricultural and Forest Entomology* 26: 201–209. doi:10.1111/afe.12607.

Anning, A. K., and B. C. McCarthy. 2013. Competition, size and age affect tree growth response to fuel reduction treatments in mixed-oak forests of Ohio. *Forest Ecology and Management* 307. Elsevier B.V.: 74–83. doi:10.1016/j.foreco.2013.07.008.

Ara, M., B. D. Pinno, and P. G. Comeau. 2024. Mixing trembling aspen and white spruce increases the understory vegetation cover and improves soil properties but effects vary with broadleaf density. *Forest Ecology and Management* 562. Elsevier B.V.: 121955. doi:10.1016/j.foreco.2024.121955.

De Araujo Pereira, A. P., P. A. M. De Andrade, D. Bini, A. Durrer, A. Robin, J. P. Bouillet, F. D. Andreote, and E. J. B. N. Cardoso. 2017. Shifts in the bacterial community composition along deep soil profiles in monospecific and mixed stands of Eucalyptus grandis and Acacia mangium. *PLoS ONE* 12: 1–15. doi:10.1371/journal.pone.0180371.

Bai, Y., Y. Zhou, Z. An, J. Du, X. Zhang, and S. X. Chang. 2022. Tree species identity and mixing ratio affected the release of several metallic elements from mixed litter in coniferous-broadleaf plantations in subtropical China. *Science of the Total Environment* 838. Elsevier B.V.: 156143. doi:10.1016/j.scitotenv.2022.156143.

Behera, M. D., A. K. Gupta, S. K. Barik, P. Das, and R. M. Panda. 2018. Use of satellite remote sensing as a monitoring tool for land and water resources development activities in an Indian tropical site. *Environmental Monitoring and Assessment* 190. Environmental Monitoring and Assessment. doi:10.1007/s10661-018-6770-8.

Bellone, D., C. Björkman, A. Schmidt, J. Gershenzon, and M. J. Klapwijk. 2021. Effect of forest stand type on host plant quality and direct and indirect effects on pine sawfly performance. *Agricultural and Forest Entomology* 23: 163–172. doi:10.1111/afe.12415.

Benomar, L., A. DesRochers, and G. R. Larocque. 2013. Comparing growth and fine root distribution in monocultures and mixed plantations of hybrid poplar and spruce. *Journal of Forestry Research* 24: 247–254. doi:10.1007/s11676-013-0348-7.

Berger, T. W., G. Köllensperger, and R. Wimmer. 2004. Plant-soil feedback in spruce (Picea abies) and mixed spruce-beech (Fagus sylvatica) stands as indicated by dendrochemistry. *Plant and Soil* 264: 69–83. doi:10.1023/B:PLSO.0000047714.43253.25.

Bolte, A., T. Rahmann, M. Kuhr, P. Pogoda, D. Murach, and & K. V Gadow. 2004. Relationships between tree dimension and coarse root biomass in mixed stands of European beech (Fagus sylvatica L.) and Norway spruce (Picea abies [L.] Karst.). *Plant and Soil* 264: 1–11. doi:https://doi.org/10.1023/B:PLSO.0000047777.23344.a3.

Bristow, M., J. K. Vanclay, L. Brooks, and M. Hunt. 2006. Growth and species interactions of Eucalyptus pellita in a mixed and monoculture plantation in the humid tropics of north Queensland. *Forest Ecology and Management* 233: 285–294. doi:10.1016/j.foreco.2006.05.019.

Brockwell, J., P. A. Mitchell, S. D. Searle, M. A. Leach, and T. E. Crews. 2011. Direct benefits of rhizobial inoculation to Acacia mearnsii De Wild, as tubed stock and in a plantation, and synergistic benefits to interplanted Eucalyptus nitens (Deane & Maiden) Maiden. *Australian Forestry* 74: 120–132. doi:10.1080/00049158.2011.10676354.

Bu, W. S., F. S. Chen, F. C. Wang, X. M. Fang, R. Mao, and H. M. Wang. 2019. The species-specific responses of nutrient resorption and carbohydrate accumulation in leaves and roots to nitrogen addition in a subtropical mixed plantation. *Canadian Journal of Forest Research* 49: 826–835. doi:10.1139/cjfr-2018-0322.

Burner, D. M., D. K. Brauer, J. L. Snider, C. A. Harrington, and P. A. Moore. 2014. Phenological responses of juvenile pecan and white oak on an upland site. *Agroforestry Systems* 88: 141–155. doi:10.1007/s10457-013-9662-5.

Calder, W. J., and S. B. St. Clair. 2012. Facilitation drives mortality patterns along succession gradients of aspen‐conifer forests. *Ecosphere* 3: 1–11. doi:10.1890/es12-00119.1.

Castagneyrol, B., M. V. Kozlov, C. Poeydebat, M. Toïgo, and H. Jactel. 2020. Associational resistance to a pest insect fades with time. *Journal of Pest Science* 93. Springer Berlin Heidelberg: 427–437. doi:10.1007/s10340-019-01148-y.

Chen, B., K. Liu, C. Wang, J. Guo, J. Lu, L. Chen, Z. Zhao, and J. Zeng. 2024. Tree allometry responses to competition and complementarity in mixed-species plantations of Betula alnoides. *Forest Ecosystems* 11. The Authors: 100207. doi:10.1016/j.fecs.2024.100207.

Chifflot, V., D. Rivest, A. Olivier, A. Cogliastro, and D. Khasa. 2009. Molecular analysis of arbuscular mycorrhizal community structure and spores distribution in tree-based intercropping and forest systems. *Agriculture, Ecosystems and Environment* 131: 32–39. doi:10.1016/j.agee.2008.11.010.

Clark, J. R., G. E. Hemery, and P. S. Savill. 2008. Early growth and form of common walnut (Juglans regia L.) in mixture with tree and shrub nurse species in southern England. *Forestry* 81: 631–644. doi:10.1093/forestry/cpn036.

Clinch, R. L., N. V. Thevathasan, A. M. Gordon, T. A. Volk, and D. Sidders. 2009. Biophysical interactions in a short rotation willow intercropping system in southern Ontario, Canada. *Agriculture, Ecosystems and Environment* 131: 61–69. doi:10.1016/j.agee.2009.01.018.

Coelho, S. R. de F., J. L. de M. Gonçalves, S. L. de M. Mello, R. M. Moreira, E. V. da Silva, and J.-P. Laclau. 2007. Crescimento, nutrição e fixação biológica de nitrogênio em plantios mistos de eucalipto e leguminosas arbóreas. *Pesquisa Agropecuária Brasileira* 42: 759–768. doi:10.1590/s0100-204x2007000600001.

Collet, C., F. Ningre, I. Barbeito, A. Arnaud, and A. Piboule. 2014. Response of tree growth and species coexistence to density and species evenness in a young forest plantation with two competing species. *Annals of Botany* 113: 711–719. doi:10.1093/aob/mct285.

Cruickshank, M. G., C. N. Filipescu, and R. N. Sturrock. 2018. The effect of stump removal and tree admixture on butt decay incidence, damage and wood density in western redcedar. *Canadian Journal of Plant Pathology* 40. Taylor & Francis: 368–377. doi:10.1080/07060661.2018.1496143.

Danilović, M., M. Kosovski, D. Gačić, D. Stojnić, and S. Antonić. 2015. Damage to residual trees and regeneration during Felling and timber extraction in mixed and pure beech stands. *Sumarski List* 139: 253–262.

Ding, H., H. Pretzsch, G. Schütze, and T. Rötzer. 2017. Size-dependence of tree growth response to drought for Norway spruce and European beech individuals in monospecific and mixed-species stands. *Plant Biology* 19: 709–719. doi:10.1111/plb.12596.

Dodet, M., C. Collet, H. Frochot, and L. Wehrlen. 2011. Tree regeneration and plant species diversity responses to vegetation control following a major windthrow in mixed broadleaved stands. *European Journal of Forest Research* 130: 41–53. doi:10.1007/s10342-010-0406-z.

Drössler, L., R. Övergaard, P. M. Ekö, P. Gemmel, and H. Böhlenius. 2015. Early development of pure and mixed tree species plantations in Snogeholm, southern Sweden. *Scandinavian Journal of Forest Research* 30. Taylor & Francis: 304–316. doi:10.1080/02827581.2015.1005127.

Fahlvik, N., E. Agestam, P. M. Ekö, and M. LindéN. 2011. Development of single-storied mixtures of Norway spruce and birch in Southern Sweden. *Scandinavian Journal of Forest Research* 26: 36–45. doi:10.1080/02827581.2011.564388.

Fanin, N., T. L. Maxwell, A. Altinalmazis-Kondylis, L. Bon, C. Meredieu, H. Jactel, M. R. Bakker, and L. Augusto. 2022. Effects of mixing tree species and water availability on soil organic carbon stocks are depth dependent in a temperate podzol. *European Journal of Soil Science* 73: 1–9. doi:10.1111/ejss.13133.

Farinha, A., B. Castagneyrol, I. van Halder, M. Branco, C. Meredieu, and H. Jactel. 2024. Reduction of damage by the exotic invasive conifer seed bug Leptoglossus occidentalis in mixed pine-birch plantations: A case of biotic resistance. *Forest Ecology and Management* 569. doi:10.1016/j.foreco.2024.122193.

Field, E., K. Schönrogge, N. Barsoum, A. Hector, and M. Gibbs. 2019. Individual tree traits shape insect and disease damage on oak in a climate-matching tree diversity experiment. *Ecology and Evolution* 9: 8524–8540. doi:10.1002/ece3.5357.

Field, E., B. Castagneyrol, M. Gibbs, H. Jactel, N. Barsoum, K. Schönrogge, and A. Hector. 2020. Associational resistance to both insect and pathogen damage in mixed forests is modulated by tree neighbour identity and drought. *Journal of Ecology* 108: 1511–1522. doi:10.1111/1365-2745.13397.

Fleischer, F., S. Eckel, I. Schmid, V. Schmidt, and M. Kazda. 2006. Point process modelling of root distribution in pure stands of Fagus sylvatica and Picea abies. *Canadian Journal of Forest Research* 36. NRC Research Press: 227–237. doi:10.1139/x05-232.

Formaglio, G., A. V. Krusche, L. Mareschal, J. P. Bouillet, J. L. de M. Gonçalves, Y. Nouvellon, J. S. Delgado-Rojas, A. Montebelo, et al. 2023. Planting nitrogen-fixing trees in tropical Eucalyptus plantations does not increase nutrient losses through drainage. *Forest Ecology and Management* 537. doi:10.1016/j.foreco.2023.120940.

Forrester, D. I., J. Bauhus, and A. L. Cowie. 2005. On the success and failure of mixed-species tree plantations: lessons learned from a model system of Eucalyptus globulus and Acacia mearnsii. *Forest Ecology and Management* 209. Elsevier: 147–155. doi:10.1016/J.FORECO.2005.01.012.

Forrester, D. I., J. Bauhus, A. L. Cowie, P. A. Mitchell, and Brockwell J. 2007. Productivity of Three Young Mixed-Species Plantations Containing ... *Forest Science* 53: 426–434.

Fruleux, A., M. B. Bogeat-Triboulot, C. Collet, and D. Bonal. 2020. Lack of effect of admixture proportion and tree density on water acquisition depth for European beech (Fagus sylvatica L.) and sycamore maple (Acer pseudoplatanus L.). *Annals of Forest Science* 77. Annals of Forest Science. doi:10.1007/s13595-020-00937-1.

Germon, A., I. A. Guerrini, B. Bordron, J. P. Bouillet, Y. Nouvellon, J. L. de Moraes Gonçalves, C. Jourdan, R. R. Paula, et al. 2018. Consequences of mixing Acacia mangium and Eucalyptus grandis trees on soil exploration by fine-roots down to a depth of 17 m. *Plant and Soil* 424: 203–220. doi:10.1007/s11104-017-3428-1.

Ghaley, B. B., and J. R. Porter. 2014. Determination of biomass accumulation in mixed belts of Salix, Corylus and Alnus species in combined food and energy production system. *Biomass and Bioenergy* 63. Elsevier Ltd: 86–91. doi:10.1016/j.biombioe.2014.02.009.

Gilbert, S., J. Martel, T. Klemola, and K. Norrdahl. 2013. Increasing vole numbers cause more lethal damage to saplings in tree monocultures than in mixed stands. *Basic and Applied Ecology* 14: 12–19. doi:10.1016/j.baae.2012.11.005.

Gosselin, M., D. Fourcin, Y. Dumas, F. Gosselin, N. Korboulewsky, M. Toïgo, and P. Vallet. 2017. Influence of forest tree species composition on bryophytic diversity in mixed and pure pine (Pinus sylvestris L.) and oak (Quercus petraea (Matt.) Liebl.) stands. *Forest Ecology and Management* 406. Elsevier: 318–329. doi:10.1016/j.foreco.2017.09.067.

Grotta, A. T., B. L. Gartner, and S. R. Radosevich. 2004. Influence of species proportion and timing of establishment on stem quality in mixed red alder - Douglas-fir plantations. *Canadian Journal of Forest Research* 34: 863–873. doi:10.1139/x03-259.

van Halder, I., B. Castagneyrol, C. Ordóñez, F. Bravo, M. del Río, L. Perrot, and H. Jactel. 2019. Tree diversity reduces pine infestation by mistletoe. *Forest Ecology and Management* 449. Elsevier: 117470. doi:10.1016/j.foreco.2019.117470.

Harper, G., K. Omari, M. Kranabetter, and P. Courtin. 2023. Assessment of a 20-year-old mixed western redcedar/red alder plantation in southwestern British Columbia. *Forest Ecology and Management* 529. Elsevier B.V.: 120737. doi:10.1016/j.foreco.2022.120737.

Hasegawa, M., A. T. Ota, D. Kabeya, T. Okamoto, T. Saitoh, and Y. Nishiyama. 2014. The effects of mixed broad-leaved trees on the collembolan community in larch plantations of central Japan. *Applied Soil Ecology* 83. Elsevier B.V.: 125–132. doi:10.1016/j.apsoil.2013.06.005.

Hedwall, P. O., E. Holmström, M. Lindbladh, and A. Felton. 2019. Concealed by darkness: How stand density can override the biodiversity benefits of mixed forests. *Ecosphere* 10. doi:10.1002/ecs2.2835.

Hicks, L. C., M. M. Rahman, M. Carnol, K. Verheyen, and J. Rousk. 2018. The legacy of mixed planting and precipitation reduction treatments on soil microbial activity, biomass and community composition in a young tree plantation. *Soil Biology and Biochemistry* 124. Elsevier: 227–235. doi:10.1016/j.soilbio.2018.05.027.

Hirai, K., J. Ferraz, S. F. Ferreira, and M. Kobayashi. 2005. Change in physical properties of ferralsols with tree planting in the Central Amazon, Brazil. *Soil Science and Plant Nutrition* 51: 761–765. doi:10.1111/j.1747-0765.2005.tb00108.x.

Hombegowda, H. C., M. Köhler, A. Röll, and D. Hölscher. 2020. Tree species and size influence soil water partitioning in coffee agroforestry. *Agroforestry Systems* 94: 137–149. doi:10.1007/s10457-019-00375-7.

Hou, L., Z. Dong, Y. Yang, D. Zhang, Shengli Zhang, and Shuoxin Zhang. 2018. Applying foliar stoichiometric traits of plants to determine fertilization for a mixed pine-oak stand in the Qinling Mountains, China. *PeerJ* 2018: 1–17. doi:10.7717/peerj.4628.

Huang, X., S. Liu, Y. You, Y. Wen, H. Wang, and J. Wang. 2017. Microbial community and associated enzymes activity influence soil carbon chemical composition in Eucalyptus urophylla plantation with mixing N2-fixing species in subtropical China. *Plant and Soil* 414: 199–212. doi:10.1007/s11104-016-3117-5.

Huber, J. A., K. May, T. Siegl, H. Schmid, G. Gerl, and K. J. Hülsbergen. 2016. Yield Potential of Tree Species in Organic and Conventional Short-Rotation Agroforestry Systems in Southern Germany. *Bioenergy Research* 9. BioEnergy Research: 955–968. doi:10.1007/s12155-016-9750-2.

Hurteau, M., H. Zald, and M. North. 2007. Species-specific response to climate reconstruction in upper-elevation mixed-conifer forests of the western Sierra Nevada, California. *Canadian Journal of Forest Research* 37: 1681–1691. doi:10.1139/X07-028.

Huuskonen, S., T. Lahtinen, J. Miina, K. Uotila, S. Bianchi, and P. Niemistö. 2023. Growth Dynamics of Young Mixed Norway Spruce and Birch Stands in Finland. *Forests* 14: 1–17. doi:10.3390/f14010056.

Hytönen, J., J. Saramäki, and P. Niemistö. 2014. Growth, stem quality and nutritional status of Betula pendula and Betula pubescens in pure stands and mixtures. *Scandinavian Journal of Forest Research* 29. Taylor & Francis: 1–11. doi:10.1080/02827581.2013.838300.

Isaac, M. E., F. Ulzen-Appiah, V. R. Timmer, and S. J. Quashie-Sam. 2007. Early growth and nutritional response to resource competition in cocoa-shade intercropped systems. *Plant and Soil* 298: 243–254. doi:10.1007/s11104-007-9362-x.

Jacob, A., and C. Leuschner. 2015. Complementarity in the use of nitrogen forms in a temperate broad-leaved mixed forest. *Plant Ecology & Diversity* 8. Taylor & Francis: 243–258. doi:10.1080/17550874.2014.898166.

Jacob, M., C. Leuschner, and F. M. Thomas. 2010. Productivity of temperate broad-leaved forest stands differing in tree species diversity. *Annals of Forest Science* 67. doi:10.1051/forest/2010005.

Jäkel, A., and M. Roth. 2004. Conversion of single-layered Scots pine monocultures into close-to-nature mixed hardwood forests: Effects on parasitoid wasps as pest antagonists. *European Journal of Forest Research* 123: 203–212. doi:10.1007/s10342-004-0030-x.

Ji, Y., P. Zhang, and H. Shen. 2023. Competition intensity affects growing season nutrient dynamics in Korean pine trees and their microhabitat soil in mixed forest. *Forest Ecology and Management* 539. Elsevier B.V.: 121018. doi:10.1016/j.foreco.2023.121018.

Jiang, X. L., R. Mao, Z. L. Li, F. S. Chen, B. Xu, J. He, Y. X. Huang, and X. M. Fang. 2023. Tree species mixture effect on extracellular enzyme kinetics varies with enzyme type and soil depth in subtropical plantations. *Plant and Soil* 493. Springer International Publishing: 267–282. doi:10.1007/s11104-023-06229-7.

Juchheim, J., M. Ehbrecht, P. Schall, C. Ammer, and D. Seidel. 2020. Effect of tree species mixing on stand structural complexity. *Forestry* 93: 75–83. doi:10.1093/forestry/cpz046.

Jucker, T., O. Bouriaud, D. Avacaritei, I. Dǎnilǎ, G. Duduman, F. Valladares, and D. A. Coomes. 2014. Competition for light and water play contrasting roles in driving diversity-productivity relationships in Iberian forests. *Journal of Ecology* 102: 1202–1213. doi:10.1111/1365-2745.12276.

Kaba, J. S., S. Zerbe, M. Agnolucci, F. Scandellari, A. A. Abunyewa, M. Giovannetti, and M. Tagliavini. 2019. Atmospheric nitrogen fixation by gliricidia trees (Gliricidia sepium (Jacq.) Kunth ex Walp.) intercropped with cocoa (Theobroma cacao L.). *Plant and Soil* 435. Plant and Soil: 323–336. doi:10.1007/s11104-018-3897-x.

Kaitaniemi, P., J. Riihimäki, J. Koricheva, and H. Vehviläinen. 2007. Experimental evidence for associational resistance against the European pine sawfly in mixed tree stands. *Silva Fennica* 41: 259–268. doi:10.14214/sf.295.

Kara, F., and S. Ö. Keleş. 2023. Tree species richness influence productivity and anatomical characteristics in mixed fir-pine-beech forests. *Plant Ecology* 224. Springer Netherlands: 479–489. doi:10.1007/s11258-023-01315-3.

Kembel, S. W., and M. R. T. Dale. 2006. Within-stand spatial structure and relation of boreal canopy and understorey vegetation. *Journal of Vegetation Science* 17: 783. doi:10.1658/1100-9233(2006)17[783:wssaro]2.0.co;2.

Khamzina, A., J. P. A. Lamers, and P. L. G. Vlek. 2009. Nitrogen fixation by Elaeagnus angustifolia in the reclamation of degraded croplands of Central Asia. *Tree Physiology* 29: 799–808. doi:10.1093/treephys/tpp017.

Kilpeläinen, H., J. Lindblad, H. Heräjärvi, and E. Verkasalo. 2011. Saw log recovery and stem quality of birch from thinnings in southern Finland. *Silva Fennica* 45: 267–282. doi:10.14214/sf.117.

Király, I., and P. Ódor. 2010. The effect of stand structure and tree species composition on epiphytic bryophytes in mixed deciduous-coniferous forests of Western Hungary. *Biological Conservation* 143: 2063–2069. doi:10.1016/j.biocon.2010.05.014.

Koupar, S. A. M., S. M. Hosseini, M. Tabari, A. Modirrahmati, A. Golchin, and F. H. Rad. 2011. Effects of pure and mixed plantations of Populus deltoides with Alnus glotinosa on growth and soil properties: A case study of Foman Region, Iran. *African Journal of Agricultural Research* 6: 5261–5265. doi:10.5897/AJAR11.163.

Kriegel, P., D. Matevski, and A. Schuldt. 2021. Monoculture and mixture-planting of non-native Douglas fir alters species composition, but promotes the diversity of ground beetles in a temperate forest system. *Biodiversity and Conservation* 30. Springer Netherlands: 1479–1499. doi:10.1007/s10531-021-02155-1.

Krišāns, O., R. Matisons, J. Vuguls, E. Bāders, S. Rust, D. Elferts, R. Saleniece, and Ā. Jansons. 2022. Regularly Planted Rather Than Natural Understory of Norway Spruce (Picea abies H. Karst.) Contributes to the Individual Stability of Canopy Silver Birch (Betula pendula Roth.). *Forests* 13. doi:10.3390/f13060942.

Kroon, J., U. Bergsten, and J. Sonesson. 2019. Increasing production value in Scots pine plantation through mixing with lodgepole pine. *Scandinavian Journal of Forest Research* 34. Taylor & Francis: 689–698. doi:10.1080/02827581.2019.1695909.

Kunert, N., and A. Mercado Cárdenas. 2015. Are mixed tropical tree plantations more resistant to drought than monocultures? *Forests* 6: 2029–2046. doi:10.3390/f6062029.

Lagache, L., E. K. Klein, A. Ducousso, and R. J. Petit. 2014. Distinct male reproductive strategies in two closely related oak species. *Molecular Ecology* 23: 4331–4343. doi:10.1111/mec.12766.

Lang, A. C., G. von Oheimb, M. Scherer-Lorenzen, B. Yang, S. Trogisch, H. Bruelheide, K. Ma, and W. Härdtle. 2014. Mixed afforestation of young subtropical trees promotes nitrogen acquisition and retention. *Journal of Applied Ecology* 51: 224–233. doi:10.1111/1365-2664.12157.

Lee Molinari, R., M. F. Bekker, B. D. St. Clair, J. Bartholomew, R. J. DeRose, S. G. Kitchen, and S. B. St. Clair. 2022. Facilitation differentially affects competitive responses of aspen and subalpine fir through stages of stand development. *Ecosphere* 13: 1–11. doi:10.1002/ecs2.3957.

Lei, J., A. Duan, W. Guo, and J. Zhang. 2024. Effects of tree species mixing and soil depth on the soil bacterial and fungal communities in Chinese fir (Cunninghamia lanceolata) plantations. *Applied Soil Ecology* 195. Elsevier B.V.: 105270. doi:10.1016/j.apsoil.2024.105270.

Lemma, B. 2012. Soil chemical properties and nutritional status of trees in pure and mixed-species stands in south Ethiopia. *Journal of Plant Nutrition and Soil Science* 175: 769–774. doi:10.1002/jpln.201100221.

Leuschner, A. G. M. J. H. F. F. M. T. C. 2009. Acidity, nutrient stocks, and organic-matter content in soils of a temperate deciduous forest with different abundance of European beech (Fagus sylvatica L.). *Journal of Plant Nutrition and Soil Science* 172.

Li, C., Y. Xu, Z. Wang, W. Zhu, and A. Du. 2023. Mixing planting with native tree species reshapes soil fungal community diversity and structure in multi-generational eucalypt plantations in southern China. *Frontiers in Microbiology* 14: 1–12. doi:10.3389/fmicb.2023.1132875.

Li, W. Q., Z. J. Wu, Y. Y. Zong, G. G. Wang, F. S. Chen, Y. Q. Liu, J. J. Li, and X. M. Fang. 2022. Tree species mixing enhances rhizosphere soil organic carbon mineralization of conifers in subtropical plantations. *Forest Ecology and Management* 516. Elsevier B.V.: 120238. doi:10.1016/j.foreco.2022.120238.

Li, Y., D. Zou, B. Ren, X. Ding, H. Bian, and J. Wang. 2016. Balancing effect of larch plantations (Larix kaempferi) on understory plant diversity in a sub tropical forest ecosystem, China. *Applied Ecology and Environmental Research* 14: 397–407. doi:10.15666/aeer/1403_397407.

Li, Y., J. Xu, H. Wang, Y. Nong, G. Sun, S. Yu, L. Liao, and S. Ye. 2021. Long-term effects of thinning and mixing on stand spatial structure: A case study of chinese fir plantations. *IForest* 14: 113–121. doi:10.3832/ifor3489-014.

Li, Z., L. Shi, Y. Kuzyakov, J. Pausch, S. Scheu, and M. M. Pollierer. 2021. The flux of root-derived carbon via fungi and bacteria into soil microarthropods (Collembola) differs markedly between cropping systems. *Soil Biology and Biochemistry* 160. Elsevier Ltd: 108336. doi:10.1016/j.soilbio.2021.108336.

Lin, N., M. Feng, H. Huang, Z. Qiu, T. Ma, and S. Chen. 2024. Effects of Thinning on Carbon Storage in a Mixed Broadleaved Plantation in a Subtropical Area of China. *Forests* 15. doi:10.3390/f15040638.

Lintunen, A., R. Sievänen, P. Kaitaniemi, and J. Perttunen. 2011. Models of 3D crown structure for Scots pine (Pinus sylvestris) and silver birch (Betula pendula) grown in mixed forest. *Canadian Journal of Forest Research* 41: 1779–1794. doi:10.1139/x11-092.

Liu, F. Y., C. J. Gao, M. Chen, and K. Li. 2018. Above- and below-ground biomass relationships of Leucaena leucocephala (Lam.) de Wit in different plant stands. *PLoS ONE* 13: 1–15. doi:10.1371/journal.pone.0207059.

Liu, J., J. Feng, H. Gao, and D. Chen. 2024. Nonlinear mixed-effect branch growth model development for planted Korean pine in Northeast China. *Trees - Structure and Function* 38. Springer Berlin Heidelberg: 409–421. doi:10.1007/s00468-024-02490-0.

Liziniewicz, M., T. Andrzejczyk, and S. Drozdowski. 2016. The effect of birch removal on growth and quality of pedunculate oak in a 21-year-old mixed stand established by row planting. *Forest Ecology and Management* 364. Elsevier B.V.: 165–172. doi:10.1016/j.foreco.2016.01.011.

Loewe-Muñoz, V., R. Del Río, C. Delard, and M. Balzarini. 2020. Mixed Pyrus pyraster and Sorbus torminalis plantations including companion species enhance high-quality timber production. *European Journal of Forest Research* 139. Springer Berlin Heidelberg: 655–664. doi:10.1007/s10342-020-01278-4.

Lu, J. K., Z. S. Li, F. C. Yang, S. K. Wang, J. F. Liang, and X. H. He. 2020. Concurrent carbon and nitrogen transfer between hemiparasite Santalum album and two N2-fixing hosts in a sandalwood plantation. *Forest Ecology and Management* 464. doi:10.1016/j.foreco.2020.118060.

Lu, S., Y. Chen, J. Sardans, and J. Peñuelas. 2024. Water and nutrient use efficiency of three tree species in monoculture and mixed stands and potential drivers in the Loess Hilly Region, China. *Plant and Soil* 496. Springer International Publishing: 657–675. doi:10.1007/s11104-023-06389-6.

Lundqvist, L., S. Spreer, and C. Karlsson. 2013. Volume production in different silvicultural systems for 85 years in a mixed Picea abies-Pinus sylvestrisforest in central Sweden. *Silva Fennica* 47: 1–14. doi:10.14214/sf.897.

Männistö, L., J. Miina, and S. Huuskonen. 2024. How to utilize natural regeneration of birch to establish mixed spruce-birch forests in Finland? *Silva Fennica* 58: 1–29. doi:10.14214/sf.23075.

Mathiasen, R. L. 2009. Comparative susceptibility of conifers to knobcone pine dwarf mistletoe. *Western North American Naturalist* 69: 42–48. doi:10.3398/064.069.0114.

Nelson, A. S., A. R. Weiskittel, and R. G. Wagner. 2014. Development of branch, crown, and vertical distribution leaf area models for contrasting hardwood species in Maine, USA. *Trees - Structure and Function* 28: 17–30. doi:10.1007/s00468-013-0926-5.

Newton, M., and E. C. Cole. 2006. Use of growth curve derivatives to illustrate acceleration and deceleration of growth in young plantations under variable competition. *Canadian Journal of Forest Research* 36: 2515–2522. doi:10.1139/X06-131.

Niccoli, F., T. Danise, M. Innangi, F. Pelleri, M. C. Manetti, G. Mastrolonardo, G. Certini, A. Fioretto, et al. 2021. Tree species composition in mixed plantations influences plant growth, intrinsic water use efficiency and soil carbon stock. *Forests* 12. doi:10.3390/f12091251.

Oak, N. R., W. Oak, B. Walnut, M. Gauthier, and D. F. Jacobs. 2010. Diameter Growth for the First 3 Years after 27.

Obando, M. F., and R. Moya. 2013. Silviculture conditions and wood properties of Samanea saman and Enterolobium cyclocarpum in 19-year-old mixed plantations. *Forest Systems* 22: 58–70. doi:10.5424/fs/2013221-02563.

Oliveira, I. R., J. P. Bouillet, J. Guillemot, C. B. Brandani, B. Bordron, C. B. Frayret, J. P. Laclau, A. V. Ferraz, et al. 2024. Changes in light use efficiency explains why diversity effect on biomass production is lower at high planting density in mixed-species plantations of Eucalyptus grandis and Acacia mangium. *Forest Ecology and Management* 554. Elsevier B.V.: 121663. doi:10.1016/j.foreco.2023.121663.

Pagano, M. C., M. N. Cabello, A. F. Bellote, N. M. Sá, and M. R. Scotti. 2008. Intercropping system of tropical leguminous species and Eucalyptus camaldulensis, inoculated with rhizobia and/or mycorrhizal fungi in semiarid Brazil. *Agroforestry Systems* 74: 231–242. doi:10.1007/s10457-008-9177-7.

Palandrani, C., and G. Alberti. 2020. Tree derived soil carbon is enhanced by tree species richness and functional diversity. *Plant and Soil* 446. Plant and Soil: 457–469. doi:10.1007/s11104-019-04381-7.

Pech-Hoil, R., M. M. Ferrer, M. Aguilar-Espinosa, R. Valdez-Ojeda, L. E. Garza-Caligaris, and R. Rivera-Madrid. 2017. Variation in the mating system of Bixa orellana L. (achiote) under three different agronomic systems. *Scientia Horticulturae* 223. Elsevier: 31–37. doi:10.1016/j.scienta.2017.05.031.

Van de Peer, T., K. Verheyen, L. Baeten, Q. Ponette, and B. Muys. 2016. Biodiversity as insurance for sapling survival in experimental tree plantations. *Journal of Applied Ecology* 53: 1777–1786. doi:10.1111/1365-2664.12721.

Van de Peer, T., K. Verheyen, Q. Ponette, N. N. Setiawan, and B. Muys. 2018. Overyielding in young tree plantations is driven by local complementarity and selection effects related to shade tolerance. *Journal of Ecology* 106: 1096–1105. doi:10.1111/1365-2745.12839.

Perot, T., A. Mårell, N. Korboulewsky, V. Seigner, and P. Balandier. 2017. Modeling and predicting solar radiation transmittance in mixed forests at a within-stand scale from tree species basal area. *Forest Ecology and Management* 390. Elsevier B.V.: 127–136. doi:10.1016/j.foreco.2017.01.023.

Plath, M., K. Mody, C. Potvin, and S. Dorn. 2011. Establishment of native tropical timber trees in monoculture and mixed-species plantations: Small-scale effects on tree performance and insect herbivory. *Forest Ecology and Management* 261. Elsevier B.V.: 741–750. doi:10.1016/j.foreco.2010.12.004.

Potvin, C., and P. Dutilleul. 2009. Neighborhood effects and size-asymmetric competition in a tree plantation varying in diversity. *Ecology* 90: 321–327.

Potvin, C., and N. J. Gotelli. 2008. Biodiversity enhances individual performance but does not affect survivorship in tropical trees. *Ecology letters* 11. England: 217–223. doi:10.1111/j.1461-0248.2007.01148.x.

Pretzsch, H., and C. Schütze. 2005. Crown allometry and growing space efficiency of Norway spruce (Picea abies [L.] Karst.) and European beech (Fagus sylvatica L.) in pure and mixed stands. *Plant Biology* 7: 628–639. doi:10.1055/s-2005-865965.

Puriņa, L., R. Matisons, Ā. Jansons, and S. Šēnhofa. 2016. Survival of European beech in the central part of Latvia 33 years since the plantation. *Silva Fennica* 50: 1–6. doi:10.14214/sf.1656.

Qu, Z. lei, B. Liu, Y. mei Zhang, L. Huang, A. gang Ming, and H. Sun. 2022. Impacts of near-natural management in eucalyptus plantations on soil bacterial community assembly and function related to nitrogen cycling. *Functional Ecology* 36: 1912–1923. doi:10.1111/1365-2435.14106.

Randriamananjara, M. A., N. J. Fenton, and A. DesRochers. 2023. How does understory vegetation diversity and composition differ between monocultures and mixed plantations of hybrid poplar and spruce? *Forest Ecology and Management* 549. Elsevier B.V.: 121434. doi:10.1016/j.foreco.2023.121434.

Redondo-Brenes, A., and F. Montagnini. 2006. Growth, productivity, aboveground biomass, and carbon sequestration of pure and mixed native tree plantations in the Caribbean lowlands of Costa Rica. *Forest Ecology and Management* 232: 168–178. doi:10.1016/j.foreco.2006.05.067.

Reverchon, F., S. H. Bai, X. Liu, and T. J. Blumfield. 2015. Tree plantation systems influence nitrogen retention and the abundance of nitrogen functional genes in the solomon islands. *Frontiers in Microbiology* 6: 1–12. doi:10.3389/fmicb.2015.01439.

Rhodes, A. C., T. Barney, and S. B. St. Clair. 2016. Stand composition, tree proximity and size have minimal effects on leaf function of coexisting aspen and subalpine fir. *PLoS ONE* 11: 1–12. doi:10.1371/journal.pone.0154395.

del Río, M., G. Schütze, and H. Pretzsch. 2014. Temporal variation of competition and facilitation in mixed species forests in Central Europe. *Plant biology (Stuttgart, Germany)* 16. England: 166–176. doi:10.1111/plb.12029.

Rötzer, T., K. H. Häberle, C. Kallenbach, R. Matyssek, G. Schütze, and H. Pretzsch. 2017. Tree species and size drive water consumption of beech/spruce forests - a simulation study highlighting growth under water limitation. *Plant and Soil* 418. Plant and Soil: 337–356. doi:10.1007/s11104-017-3306-x.

Rouhi-Moghaddam, E., S. M. Hosseini, E. Ebrahimi, M. Tabari, and A. Rahmani. 2008. Comparison of growth, nutrition and soil properties of pure stands of Quercus castaneifolia and mixed with Zelkova carpinifolia in the Hyrcanian forests of Iran. *Forest Ecology and Management* 255: 1149–1160. doi:10.1016/j.foreco.2007.10.048.

Rugani, B., K. Golkowska, I. Vázquez-Rowe, D. Koster, E. Benetto, and P. Verdonckt. 2015. Simulation of environmental impact scores within the life cycle of mixed wood chips from alternative short rotation coppice systems in Flanders (Belgium). *Applied Energy* 156. Elsevier Ltd: 449–464. doi:10.1016/j.apenergy.2015.07.032.

Rumberger, M. D., B. Münzenberger, O. Bens, F. Ehrig, P. Lentzsch, and R. F. Hüttl. 2004. Changes in diversity and storage function of ectomycorrhiza and soil organoprofile dynamics after introduction of beech into Scots pine forests. *Plant and Soil* 264: 111–126. doi:10.1023/B:PLSO.0000047793.14857.4f.

Šálek, L., and R. Sloup. 2012. Economic evaluation of proposed pure and mixed stands in Central Vietnam highlands. *Journal of Agriculture and Rural Development in the Tropics and Subtropics* 113: 21–29.

Sanaei, A., H. Herrmann, L. Alshaabi, J. Beck, O. Ferlian, K. W. Fomba, S. Haferkorn, M. van Pinxteren, et al. 2023. Changes in biodiversity impact atmospheric chemistry and climate through plant volatiles and particles. *Communications Earth and Environment* 4. Springer US: 1–12. doi:10.1038/s43247-023-01113-9.

Sayyad, E., S. M. Hosseini, J. Mokhtari, R. Mahdavi, S. G. Jalali, M. Akbarinia, and M. Tabari. 2006. Comparison of growth, nutrition and soil properties of pure and mixed stands of Populus deltoides and Alnus subcordata. *Silva Fennica* 40: 27–35. doi:10.14214/sf.350.

Schuldt, A., P. Huke, J. Glatthorn, J. Hagge, B. Wildermuth, and D. Matevski. 2022. Tree mixtures mediate negative effects of introduced tree species on bird taxonomic and functional diversity. *Journal of Applied Ecology* 59: 3049–3060. doi:10.1111/1365-2664.14300.

Schweier, J., C. Arranz, C. A. Nock, D. Jaeger, and M. Scherer-Lorenzen. 2019. Impact of Increased Genotype or Species Diversity in Short Rotation Coppice on Biomass Production and Wood Characteristics. *Bioenergy Research* 12. BioEnergy Research: 497–508. doi:10.1007/s12155-019-09997-2.

da Silva, E. V., J. L. de Moraes Gonçalves, S. R. de Freitas Coelho, R. M. e Moreira, S. L. de Miranda Mello, J. P. Bouillet, C. Jourdan, and J. P. Laclau. 2009. Dynamics of fine root distribution after establishment of monospecific and mixed-species plantations of Eucalyptus grandis and Acacia mangium. *Plant and Soil* 325: 305–318. doi:10.1007/s11104-009-9980-6.

Simončič, T., A. Bončina, K. Jarni, and M. Klopčič. 2019. Assessment of the long-term impact of deer on understory vegetation in mixed temperate forests. *Journal of Vegetation Science* 30: 108–120. doi:10.1111/jvs.12702.

Singhal, V., T. Roy, C. Singh, and J. Ghosh. 2021. Effect of incubation time, litter diversity and species richness on decomposition dynamics of tree species from western Himalayas. *Catena* 203. Elsevier B.V.: 105281. doi:10.1016/j.catena.2021.105281.

Soares, G. M., L. D. Silva, A. R. Higa, A. A. Simon, and J. F. B. de S. José. 2018. Crescimento de Acacia mearnsii De Wild e Eucalyptus globulus Labill em monocultivos e consórcios com linhas simples e duplas de plantio. *Scientia Forestalis* 46: 571–581. doi:10.18671/scifor.v46n120.06.

Soares, G. M., L. D. Silva, A. R. Higa, A. A. Simon, and J. F. B. De São José. 2021. Artificial neural networks (Ann) for height estimation in a mixed-species plantation of eucalyptus globulus labill and acacia mearnsii de wild. *Revista Arvore* 45: 1–9. doi:10.1590/1806-908820210000012.

Steinbauer, M. J., M. W. Short, and S. Schmidt. 2006. The influence of architectural and vegetational complexity in eucalypt plantations on communities of native wasp parasitoids: Towards silviculture for sustainable pest management. *Forest Ecology and Management* 233: 153–164. doi:10.1016/j.foreco.2006.06.019.

Stille, L., E. Smeets, B. Wicke, R. Singh, and G. Singh. 2011. The economic performance of four (agro-) forestry systems on alkaline soils in the state of Haryana in India. *Energy for Sustainable Development* 15. International Energy Initiative: 388–397. doi:10.1016/j.esd.2011.08.002.

Tang, X. Y., M. Xu, X. D. Wang, and J. Zhang. 2024. Tree species composition affects litter eco-hydrological function in Pinus massoniana conifer-broadleaf mixed forest stands in southwest China. *Hydrological Processes* 38: 1–11. doi:10.1002/hyp.15104.

Valor, T., E. Ormeño, and P. Casals. 2017. Temporal effects of prescribed burning on terpene production in Mediterranean pines. *Tree Physiology* 37: 1622–1636. doi:10.1093/treephys/tpx069.

Vehviläinen, H., and J. Koricheva. 2006. Moose and vole browsing patterns in experimentally assembled pure and mixed forest stands. *Ecography* 29: 497–506. doi:10.1111/j.0906-7590.2006.04457.x.

Vehviläinen, H., J. Koricheva, and K. Ruohomäki. 2008. Effects of stand tree species composition and diversity on abundance of predatory arthropods. *Oikos* 117: 935–943. doi:10.1111/j.0030-1299.2008.15972.x.

Viera, M., M. V. Schumacher, E. V. Liberalesso, and R. Rodríguez-Soalleiro. 2015. Mixed and monospecific stands of eucalyptus and Blackwattle. II - Fine root biomass density. *Cerne* 21: 209–217. doi:10.1590/01047760201521021255.

Waithaisong, K., A. Robin, L. Mareschal, J. P. Bouillet, J. P. Laclau, P. Deleporte, J. L. de M. Gonçalves, J. M. Harmand, et al. 2020. Introducing N2-fixing trees (Acacia mangium) in eucalypt plantations rapidly modifies the pools of organic P and low molecular weight organic acids in tropical soils. *Science of the Total Environment* 742. Elsevier B.V.: 140535. doi:10.1016/j.scitotenv.2020.140535.

Wambsganss, J., F. Beyer, G. T. Freschet, M. Scherer-Lorenzen, and J. Bauhus. 2021. Tree species mixing reduces biomass but increases length of absorptive fine roots in European forests. *Journal of Ecology* 109: 2678–2691. doi:10.1111/1365-2745.13675.

Wang, H., Z. Song, Jingxin Wang, Y. Yang, Jian Wang, and S. Liu. 2022. The quadratic relationship between tree species richness and topsoil organic carbon stock in a subtropical mixed-species planted forest. *European Journal of Forest Research* 141. Springer Berlin Heidelberg: 1151–1161. doi:10.1007/s10342-022-01498-w.

Wang, L., X. Zhou, Y. Wen, and D. Sun. 2022. Non-Additive Effects of Mixing Eucalyptus and Castanopsis hystrix Trees on Carbon Stocks under an Eco-Silviculture Regime in Southern China. *Forests* 13: 1–17. doi:10.3390/f13050733.

Wang, M., L. Baeten, F. Van Coillie, K. Calders, K. Verheyen, Q. Ponette, H. Blondeel, B. Muys, et al. 2024. Tree species identity and interaction determine vertical forest structure in young planted forests measured by terrestrial laser scanning. *Forest Ecosystems* 11. The Authors: 100196. doi:10.1016/j.fecs.2024.100196.

Wang, S., W. Zhang, and F. Sanchez. 2010. Relating net primary productivity to soil organic matter decomposition rates in pure and mixed Chinese fir plantations. *Plant and Soil* 334: 501–510. doi:10.1007/s11104-010-0400-8.

Wang, W., D. Chen, Q. Zhang, X. Sun, and S. Zhang. 2020. Effects of mixed coniferous and broad-leaved litter on bacterial and fungal nitrogen metabolism pathway during litter decomposition. *Plant and Soil* 451: 307–323. doi:10.1007/s11104-020-04523-2.

Westaway, S., C. Kling, and J. Smith. 2018. A comparison of the performance of three sward mixtures sown under trees in a silvopoultry system in the UK. *Agroforestry Systems* 92. Springer Netherlands: 1009–1018. doi:10.1007/s10457-017-0142-1.

Willis, J. L., and M. A. Blazier. 2022. Competition intensity varies with hardwood species identity and constrains stand-level productivity in southeastern pine–hardwood mixtures compared to loblolly pine monocultures. *Canadian Journal of Forest Research* 52: 1439–1458. doi:10.1139/cjfr-2022-0125.

Wolz, K. J., B. E. Branham, and E. H. DeLucia. 2018. Reduced nitrogen losses after conversion of row crop agriculture to alley cropping with mixed fruit and nut trees. *Agriculture, Ecosystems and Environment* 258. Elsevier: 172–181. doi:10.1016/j.agee.2018.02.024.

Xu, H., Y. You, Y. Wang, G. Gao, A. Ming, and X. Huang. 2024. Introducing N2-fixing tree species into Eucalyptus plantations increases organic phosphorus transformation but decreases its accumulation within aggregates in subtropical China. *Plant and Soil* 504: 191–208. doi:10.1007/s11104-024-06663-1.

Xu, Y., C. Li, W. Zhu, Z. Wang, L. Wu, and A. Du. 2022. Effects of enrichmemt planting with native tree species on bacterial community structure and potential impact on Eucalyptus plantations in southern China. *Journal of Forestry Research* 33. Springer Singapore: 1349–1363. doi:10.1007/s11676-021-01433-6.

Yan, L., Y. Wen, X. Zhou, H. Li, W. Wu, V. S. J. Sunoj, H. Lambers, and P. M. Finnegan. 2024. Adding Castanopsis hystrix to a Pinus massoniana plantation changed leaf phosphorus and nitrogen investment and soil nitrogen concentrations. *Plant and Soil* 496: 31–49. doi:10.1007/s11104-023-06097-1.

Yang, L., P. Wang, and C. Kong. 2010. Effect of larch (Larix gmelini Rupr.) root exudates on Manchurian walnut (Juglans mandshurica Maxim.) growth and soil juglone in a mixed-species plantation. *Plant and Soil* 329: 249–258. doi:10.1007/s11104-009-0149-0.

Yang, Q., and X. Chen. 2023. Plant phylogenetic relatedness and herbivore specialization interact to determine pest biocontrol efficiency in mixed plantations. *Frontiers in Ecology and Evolution* 11. doi:10.3389/fevo.2023.1187996.

Zalloni, E., G. Battipaglia, P. Cherubini, M. Saurer, and V. De Micco. 2019. Wood growth in pure and mixed Quercus ilex l. Forests: Drought influence depends on site conditions. *Frontiers in Plant Science* 10: 1–14. doi:10.3389/fpls.2019.00397.

Zhang, B., Q. Xu, S. Liu, B. Gu, D. Gao, T. Wang, M. Sui, H. Zuo, et al. 2024. Unraveling the effects of plant and soil properties on tree water absorption in pure and mixed forests across subtropical China. *Agricultural and Forest Meteorology* 353. Elsevier B.V.: 110078. doi:10.1016/j.agrformet.2024.110078.

Zhang, H., X. Li, S. Wang, C. Jiang, Y. Cui, R. Fan, Y. Lan, Q. Zhang, et al. 2024. Tree–litter–soil system C:N:P stoichiometry and tree organ homeostasis in mixed and pure Chinese fir stands in south subtropical China. *Frontiers in Forests and Global Change* 7: 1–12. doi:10.3389/ffgc.2024.1293439.

Zhang, J., M. D. Busse, D. H. Young, G. O. Fiddler, J. W. Sherlock, and J. D. TenPas. 2017. Aboveground biomass responses to organic matter removal, soil compaction, and competing vegetation control on 20-year mixed conifer plantations in California. *Forest Ecology and Management* 401: 341–353. doi:10.1016/j.foreco.2017.07.023.

Zhang, S., D. Landuyt, K. Verheyen, and P. De Frenne. 2022. Tree species mixing can amplify microclimate offsets in young forest plantations. *Journal of Applied Ecology* 59: 1428–1439. doi:10.1111/1365-2664.14158.

Zhao, D., and M. Kane. 2012. Differences in growth dynamics of loblolly and slash pine plantations in the southeastern United States. *Forest Ecology and Management* 281. Elsevier B.V.: 84–92. doi:10.1016/j.foreco.2012.06.027.

Zou, X., Z. Miao, Y. Hao, X. Liu, L. Dong, and F. Li. 2024. Effects of tree vigor, competition and stand conditions on branch diameter for mixed plantations of Fraxinus mandshurica Rupr. and Larix olgensis Henry in Northeast China. *European Journal of Forest Research* 143. Springer Berlin Heidelberg: 1165–1180. doi:10.1007/s10342-024-01681-1.
